# Lipopolysaccharide pretreatment increases the sensitivity of the TRPV1 channel and promotes an anti-inflammatory phenotype of capsaicin-activated macrophages

**DOI:** 10.1186/s12950-024-00391-0

**Published:** 2024-05-24

**Authors:** Daniel Vašek, Natálie Fikarová, Vendula Nagy Marková, Ondřej Honc, Lenka Pacáková, Bianka Porubská, Veronika Somova, Jiří Novotný, Barbora Melkes, Magdaléna Krulová

**Affiliations:** 1 Department of Cell Biology, Faculty of Science, Charles University, Vinicna 7, Prague, 2, 128 43 Czech Republic; 2 Department of Physiology, Faculty of Science, Charles University, Vinicna 7, Prague, 2, 128 43 Czech Republic; 3 Department of Parasitology, Faculty of Science, Charles University, Vinicna 7, Prague, 2, 128 43 Czech Republic

**Keywords:** TRPV1, Macrophages, Capsaicin, Inflammation

## Abstract

**Background:**

The transient receptor potential vanilloid 1 (TRPV1) is well-established in neuronal function, yet its role in immune reactions remains enigmatic. The conflicting data on its inflammatory role, suggesting both pro-inflammatory and anti-inflammatory effects upon TRPV1 stimulation in immune cells, adds complexity. To unravel TRPV1 immunomodulatory mechanisms, we investigated how the TRPV1 agonist capsaicin influences lipopolysaccharide (LPS)-induced pro-inflammatory macrophage phenotypes.

**Results:**

Changes in the surface molecules, cytokine production, and signaling cascades linked to the phenotype of M1 or M2 macrophages of the J774 macrophage cell line and bone marrow-derived macrophages, treated with capsaicin before or after the LPS-induced inflammatory reaction were determined. The functional capacity of macrophages was also assessed by infecting the stimulated macrophages with the intracellular parasite *Leishmania mexicana*.

**Conclusion:**

Our findings reveal that TRPV1 activation yields distinct macrophage responses influenced by the inflammatory context. LPS pre-treatment followed by capsaicin activation prompted increased calcium influx, accompanied by a shift toward an anti-inflammatory M2b-like polarization state.

**Supplementary Information:**

The online version contains supplementary material available at 10.1186/s12950-024-00391-0.

## Introduction

The transient receptor potential vanilloid 1 (TRPV1) is a non-selective cation channel associated with pain signaling and neurogenic inflammation [1, 2]. Nevertheless, accumulating evidence suggests that TRPV1 is also involved in many other processes such as insulin sensitivity, airway hypersensitivity, urinary bladder functions, and notably, regulation of the immune response [3–7]. Activation pathways of nociceptive TRPV channels in neurons have been widely studied [8–10], but their exact role in immune cells remains largely unknown, and moreover published results are often contradictory.

Various endogenous and exogenous molecules interacting with the TRPV1 have been identified. Namely inflammatory molecules [9], cytokines, hormones [5, 11], opioids [12], growth factors, and miRNAs [13], along with mechanical stimuli, UV radiation, decreased pH, and increased temperature [14]. Among numerous exogenous modulators, resiniferatoxin, capsaicin or its antagonist capsazepine are well established [15]. The broad spectrum of TRPV1 modulating molecules indicates a complex regulation of its action, and, indeed, TRPV1 has been demonstrated to be an active player in the pleiotropic immune network. Regarding the role of TRPV1 in the immune system, its activation was initially associated with the induction of an inflammatory response [16]. However, later studies showed that TRPV1 stimulation at the site of ongoing inflammation suppressed the pro-inflammatory effect or even led to the production of anti-inflammatory cytokines. In addition, TRPV1 antagonists were able to suppress the release of inflammatory molecules in a LPS-mediated inflammation in murine macrophages [17] and TRPV1 knockout mice showed an aggravated inflammatory response after LPS injection [18].

The expression of TRPV1 has been confirmed in various immune cells, including T lymphocytes, macrophages, natural killer cells, dendritic cells, and neutrophils [10]. Unfortunately, the role of TRPV1 activation in individual immune populations remains controversial. For example, activated TRPV1 channel regulates the level of intracellular calcium in T lymphocytes, playing a key role in TCR signaling, and possibly participate in T cell development in the thymus [19, 20]. On the contrary, oral administration of capsaicin suppressed the activation of autoreactive T cells in the pancreatic lymph nodes and protected mice from the development of type 1 diabetes. This suppression was specifically mediated through macrophages [21]. Furthermore, the contribution of TRP channels to the macrophage polarization into the alternative M2 phenotype has been documented [22]. An important role for antigen-presenting cells was shown in mice with TRPV1 gain-of-function mutation. Duo et al. (2020) demonstrated that a constitutively active TRPV1 channel exacerbated DSS-induced colitis, and consequently, patients with IBD showed significantly enhanced expression of TRPV1 protein in infiltrating immune cells in the lamina propria of the inflamed colon [23]. Yet another important role of TRPV1 function in cancer growth and metastasis has also been documented; it can promote or suppress cancer cell death, depending on the type and environment [24]. The crucial role of macrophages in cancerogenesis has been shown in a mouse model of colorectal carcinoma, where gain of function of TRPV1 increased tumor incidence and burden. The effect of TRPV1 overexpression on the M1/ M2 pathways was confirmed, creating a deleterious microenvironment for tumorigenesis [25].

Together, the current research confirmed TRPV1 as an important physiological and pathophysiological molecule that significantly regulates macrophage function. To clarify the role of TRPV1 in the inflammatory response, in the present study, macrophages were stimulated with capsaicin simultaneously, before or after the LPS-induced inflammatory response and signaling cascades and various markers and functional properties associated with the phenotype of M1 or M2 macrophages were determined.

## Materials and methods

### Culture and stimulation of J774 and BMDMS

Experiments were carried out using the J774.2 murine macrophage cell line originally derived from BALB/c mouse (J774; Sigma-Aldrich, St. Louis, MO, USA) or bone marrow (BM)-derived macrophage differentiated from BM isolated from BALB/c mice of both sexes, aged 8–12 weeks (AnLab, Prague, Czech Republic). BM was cultured in Dulbecco’s Modified Eagle’s Medium – high glucose (DMEM; Sigma-Aldrich) with 20% M-CSF-conditioned medium (M-CSF-conditioned medium was collected from L929 M-CSF cell line) for one week; afterwards, macrophages were used in experiments. J774 (2,5 × 10^5^ cells/ml) or BM-derived macrophages (5 × 10^5^ cells/ml) were cultured in a volume of 1 ml DMEM supplemented with 10% fetal bovine serum (FBS; Sigma-Aldrich), antibiotics (100 µg/ml streptomycin, 100 U/ml penicillin) and 10 mM HEPES buffer (hereafter called ‘complete DMEM’) in 24-well tissue culture plates (Nunc, Roskilde, Denmark). Capsaicin (10 µM; Sigma-Aldrich) was used for TRPV1-dependent activation as is routinely used [26, 27]. Lipopolysaccharide (LPS; 1,25 µg/ml; #L2880; Sigma-Aldrich) was used for the induction of immune responses. The concentrations and times of stimulation were tested and selected according to calibration and kinetic experiments. The preincubation was set for 4 h and the stimulation varied among methods. In detail, 5 minutes for the study of MAP kinases phosphorylation, 10 min for the translocation of ERK1/2 into the nucleus, and 24 h for microscopy, PCR, ELISA, and flow cytometry (48 h for LIGHT, CD206, Mgl2 and CD163).

### Immunostaining of J774

Stimulated J774 cells (1,5 × 10^3^ cells/ml) were cultured in 24-well tissue culture plate for 24 h. Cells were washed in PBS and fixed with 4% paraformaldehyde in PBS for 10 min, then washed in PBS and stained with Wheat Germ Agglutinin 647 (1:200; Thermo Fisher Scientific, Waltham, MA, USA) for 10 min at 37 °C. Subsequently, cells were washed in PBS, permeabilized with 0,1% Triton X-100® in PBS and blocked with 1% BSA and 10% donkey serum for 1 h at 37 °C. The cells were then washed in PBS and stained for 1 h at 37 °C with rabbit anti-mouse TRPV1 antibody (ACC-030, 1:200; Alomone Labs, Jerusalem, Israel), which has been validated using a KO mouse model [28]. After washing in PBS, cells were stained with the secondary donkey anti-rabbit IgG antibody Alexa Fluor Plus 488 (1:300; Thermo Fisher). Sample mounting with nuclei staining was performed using Ibidi mounting medium with DAPI (IBIDI, Gräfelfing, Germany). The samples were observed with Carl Zeiss LSM 880 NLO microscope (Zeiss, Oberkochen, Germany). Huygens deconvolution was performed on the high-resolution confocal images.

### PCR

Total RNA was isolated from the cultured cells using TRIreagent® (Molecular Research Center, Cincinnati, OH, USA) according to the manufacturer’s instructions. Reverse transcription was performed with SuperScript™ IV Reverse Transcriptase (Thermo Fisher) according to the manufacturer’s instructions, including RNaseOUT™ Recombinant Ribonuclease Inhibitor (Thermo Fisher) and Random Hexamer Primer (Thermo Fisher). RT-PCR was performed by PPP Master Mix (Top-Bio, Vestec, Czech Republic) according to the manufacturer’s instructions. Quantitative PCR was performed by using HOT FIREPol® EvaGreen® qPCR Mix Plus (Solis BioDyne, Tartu, Estonia) according to the manufacturer’s instructions and measured by LightCyler480 (Roche, Basel, Switzerland). *Gapdh* and *Actb* were used as housekeeping genes. The primers are detailed in Supplementary Table S1.

### SDS-page electrophoresis and western blot

J774 samples were sonicated and solubilized in Laemmli buffer. The proteins were separated using SDS-Page Electrophoresis and transferred to the nitrocellulose membrane. The membrane was blocked in 5% non-fat dry milk in TBS (10mM Tris, 150mM NaCl; pH 8.0) for 30 min. Specific primary antibodies were diluted in 1% non-fat dry milk or BSA in TBS. The nitrocellulose membrane was incubated with the following primary antibodies against extracellular signal-regulated kinase (ERK)1/2 (137F5; Cell Signaling, Danvers, MA, USA), p-ERK1/2 (197G2; Cell Signaling), p38 (sc-535; Santa Cruz, Dalas, TX, USA) or p-p38 (D3F9; Cell Signaling), and gently rocked at 4 °C overnight, followed by three 10 min washes in TBS containing 0,3% Tween-20. β-Actin (sc-47778, Santa Cruz) was used as a protein-loading control. Subsequently, the membrane was incubated with appropriate HRP-conjugated secondary antibodies for 1 h at RT and followed by three 10 min washes as mentioned above. Protein bands were visualized using enhanced chemiluminescence according to the manufacturer’s instructions and developed using the ChemiDoc Imaging System (BioRad; USA). The developed images were analyzed using ImageLab software.

### FLUO4-NW calcium measurement

Calcium influx was determined using FLUO4-NW dye (Thermo Fisher) according to the manufacturer’s instructions. Briefly, J774 cells were seeded in the Poly-L Lysine coated 96 black well plate with a clear bottom. After 24 h, the dye was diluted in an assay buffer containing 2,5 mM Probenecid. Medium was removed, and cells were incubated with the dye for 30 min at 37 °C in a humified atmosphere with 5% CO_2_. Calcium response was measured at 37 °C using the ClarioStar Plus microplate reader. Excitation was set at 494 nm and emission was measured at 516 (± 30) nm. The signal was measured every 1 s. After 3 s of measurement, the appropriate ligand was automatically added by the machine using a pump. Data of the FLUO4 fluorescence changes were normalized to the baseline (before the addition of ligand) and calculated as a fold increase over the baseline.

### ERK1/2 nuclear translocation

Cultured cells were washed in ice cold PBS, fixed (1% PFA, 20 min, RT), permeabilized (100% methanol, 15 min on ice) and stained with the following antibodies: anti-p44/42 MAPK (ERK1/2; 137F5; Cell Signaling); secondary antibody Goat anti-Rabbit IgG (A-11,012; Alexa Fluor 594; Thermo Fisher) at times and concentrations recommended by the manufacturer. For cell nuclei visualization, Hoechst 33258 fluorescent dye (Sigma-Aldrich) was used 5 min before measurement. Analysis of the samples was performed using the Amnis® ImageStream® Mk II imaging cytometer (Luminex Corporation, Austin, Tx, USA). The data obtained were then analyzed using Ideas software (Luminex Corporation) and its built-in module for the analysis of nuclear translocation. Briefly – after gating the cells based on their size and aspect ratio (singlets determination) and focus gradient, the shape of each cell and its nucleus was determined by the built-in algorithm. The correlation of Hoechst 33258 (nucleus) and AF 594 (ERK1/2) signal intensity within the cell was evaluated, and the Similarity Median (SM) parameter was calculated. The area containing cells with signs of translocation was gated using the SM parameter, and cells within this gate were scored as „ERK translocated“. Representative images of the cells, dot plots, and gates are shown in Supplementary Fig. S1.

### Staining of reactive oxygen species, intracellular nitric oxide and membrane potential of mitochondria

J774 were stained for the detection of ROS production using 2′,7′-Dichlorofluorescin diacetate (DCFDA; 15 µM; Sigma-Aldrich), for intracellular nitric oxide (NO) using diaminofluorescein-2 diacetate (DAF-2 DA; 2,5 µM; Abcam, Cambridge, UK) or for mitochondrial membrane potential using MitoTracker™ Red CMXRos (100 nM; Thermo Fisher) according to the manufacturer’s instructions for 30 min at 37 °C. Cells were harvested and washed in PBS/0.5% BSA. A total of 50 000 cells were analyzed after the exclusion of dead cells and debris. Five minutes before measurement, Hoechst 33258 fluorescent dye was added and used to exclude dead cells. Data were collected using the LSR II cytometer and analyzed using GateLogic 400.2 A software. Representative dot plots and histograms illustrating the gating strategy are shown in Supplementary Figure S2.

### Characterization of surface markers by flow cytometry

Cultured cells were harvested and washed in PBS/0.5% BSA and incubated for 30 min on ice with Alexa Fluor 700 labeled anti-CD45 monoclonal antibody (mAb) (clone 30-F11; BioLegend), APC labeled anti-CD11b mAb (M1/70; BioLegend), FITC labeled anti-H-2Kb/H-2Db mAb (MHCI; 28-8-6, BioLegend), FITC labeled anti-I-A / I-E mAb (MHCII; M5/114.15.2, BioLegend), FITC labeled anti-CD80 mAb (16-10A1; BioLegend), PE labeled anti-CD86 mAb (PO3; BioLegend), PE/Cy7 labeled anti-CD301b mAb (URA-1; BioLegend) or PE labeled polyclonal Ab anti-TNFSF14 (LIGHT; Bioss Antibodies, Woburn, MA, USA). Unstained cells were used as controls. A total of 50 000 cells were analyzed after exclusion of dead cells and debris. Five minutes before measurement, Hoechst 33258 fluorescent dye (Sigma-Aldrich) was added and used to exclude dead cells. Data were collected using the LSR II cytometer (BD Bioscience, Franklin Lakes, NJ, USA) and analyzed using GateLogic 400.2 A software (Invai, Mentone, Australia). Representative dot plots and histograms illustrating the gating strategy are shown in Supplementary Fig. S3.

### Functional test with J774 and T helper cells

Naive CD4^+^ T helper cells were isolated from the spleen of BALB/c mice using FACS Aria II (BD Bioscience). Red blood cells were removed from the splenic suspension using ACK buffer and splenocytes were stained with Alexa Fluor 700 labeled anti-CD4 mAb (GK1.5; BioLegend), dead cells were excluded by propidium iodide (Exbio, Vestec, Czech Republic). J774 cells (2 × 10^4^ cells/ml) were cultivated for 4 hours with or w/o stimulation. Then CD4^+^ T helper cells (2 × 10^5^ cells/ml) were added together with stimulation with LPS or capsaicin. After 72 h, the proliferation expression of Ki67 was detected using flow cytometry. Briefly, cells were harvested, washed with PBS/0.5% BSA and incubated for 30 min on ice with Alexa Fluor 700 labeled anti-CD4 mAb (GK1.5; BioLegend), APC labeled anti-CD11b mAb (M1/70; BioLegend) and LIVE/DEAD™ Fixable Violet Dead Cell Stain Kit (Thermo Fisher). Cells were then fixed and permeabilized using a Foxp3 Staining Buffer Set (Thermo Fisher) according to manufacturer’s instructions. Subsequently, cells were stained intracellularly for 30 min with PE labeled anti-Ki67 mAb (SolA15; Thermo Fisher). All events were analyzed after the exclusion of dead cells and debris. Data were collected using the LSR II cytometer and analyzed using GateLogic 400.2 A. Representative dot plots illustrating the gating strategy are shown in Supplementary Fig. S4.

### Intracellular detection of cytokines and markers

To analyze intracellular cytokine production, Phorbol 12-Myristate 13-Acetate (PMA; 20 ng/ml; Sigma-Aldrich), ionomycin (500 ng/ml; Sigma-Aldrich) and Brefeldin A (5 µg/ml; Thermo Fisher) were added to the cultures for at least 4 h of the incubation period. Cells were harvested, washed in PBS/0,5% BSA and incubated for 30 min on ice with Alexa Fluor 700 labeled anti-CD45 mAb (clone 30-F11; BioLegend), APC labeled anti-CD11b mAb (M1/70; BioLegend) and LIVE/DEAD™ Fixable Violet Dead Cell Stain Kit (Thermo Fisher) for staining dead cells. Cells were then fixed and permeabilized using a Fixation and Permeabilization Kit (Thermo Fisher) according to the manufacturer’s instructions. Cells were then intracellularly stained for 30 min with PE labeled anti-TNFα mAb (TN3-19.12; Thermo Fisher), APC labeled anti-IL-6 mAb (MP5-20F3; BioLegend), FITC labeled anti-IL-1β Pro-form mAb (NJTEN3; Thermo Fisher), FITC labeled anti-CD206 mAb (C068C2; BioLegend) or PE labeled anti-CD163 mAb (TNKUPJ; Thermo Fisher). A total of 50 000 cells were analyzed after exclusion of dead cells and debris. Data were collected using the LSR II cytometer and analyzed using GateLogic 400.2 A software. Representative dot plots illustrating the gating strategy are shown in Supplementary Fig. S5.

### ELISA cytokine detection

Cell supernatants were harvested after 24 h and the concentration of cytokines IL-1β, IL-6 and TNFα was measured by ELISA according to the manufacturer’s instructions (R&D Systems, Minneapolis, MN, USA).

### Leishmania Cell Culture & Macrophage Infection

The promastigotes labelled with GFP *L. mexicana* (MNYC / BZ / 62 / M379) were cultured in M199 (Sigma-Aldrich) supplemented with 10% FBS, 1% BME vitamins (Sigma-Aldrich), 0.5% sterile human urine and 0.1% amikacin (Sigma-Aldrich) at 23 °C. The low passage of parasites was used for the experiments.

Stimulated J774 cells were cultured in complete DMEM medium on a 6-well tissue culture plate for 24 h. Cells were harvested and counted. 5 × 10^4^ cells were plated in 12-well tissue culture plate in 2 ml complete DMEM and infected with *L. mexicana* promastigotes at a stationary phase of growth with the ratio of 6 parasite promastigotes per 1 macrophage. 72 h after infection, cells were analyzed by flow cytometry. The number of amastigotes was counted by hemocytometer as described [29]. For flow cytometry, cells were harvested and washed in PBS/0,5% BSA. All events were analyzed after the exclusion of dead cells and debris. 5 min before measurement, Hoechst 33258 fluorescent dye was added to exclude dead cells. Data were collected using the LSR II cytometer and analyzed using GateLogic 400.2 A software. Representative dot plots and histograms illustrating the gating strategy are shown in Supplementary Fig. S6.

### Statistical analysis

For statistical analysis, the program The Prism (GraphPad Software, San Diego, CA, USA) was used. Data are shown as mean ± standard deviation (SD) or as boxes and whiskers. Lines inside the boxes represent the median, and whiskers represent the minimum and maximum values. In detail, 3 samples/group for nuclear translocation and for *L. mexicana* infection, 4 samples/group for ELISA, 5 samples/group for flow cytometry and qPCR, and 6 samples/group for western blot analysis. The statistical significance of differences between individual groups was calculated using ordinary One-Way analysis of variance (ANOVA) followed by Tukey’s post hoc test for multiple comparisons. Two-way ANOVA followed by Tukey’s post hoc test was calculated for calcium measurement. A value of *p* < 0.05 was considered statistically significant.

## Results

### TRPV1 expression in murine macrophages

**Fig. 1:**
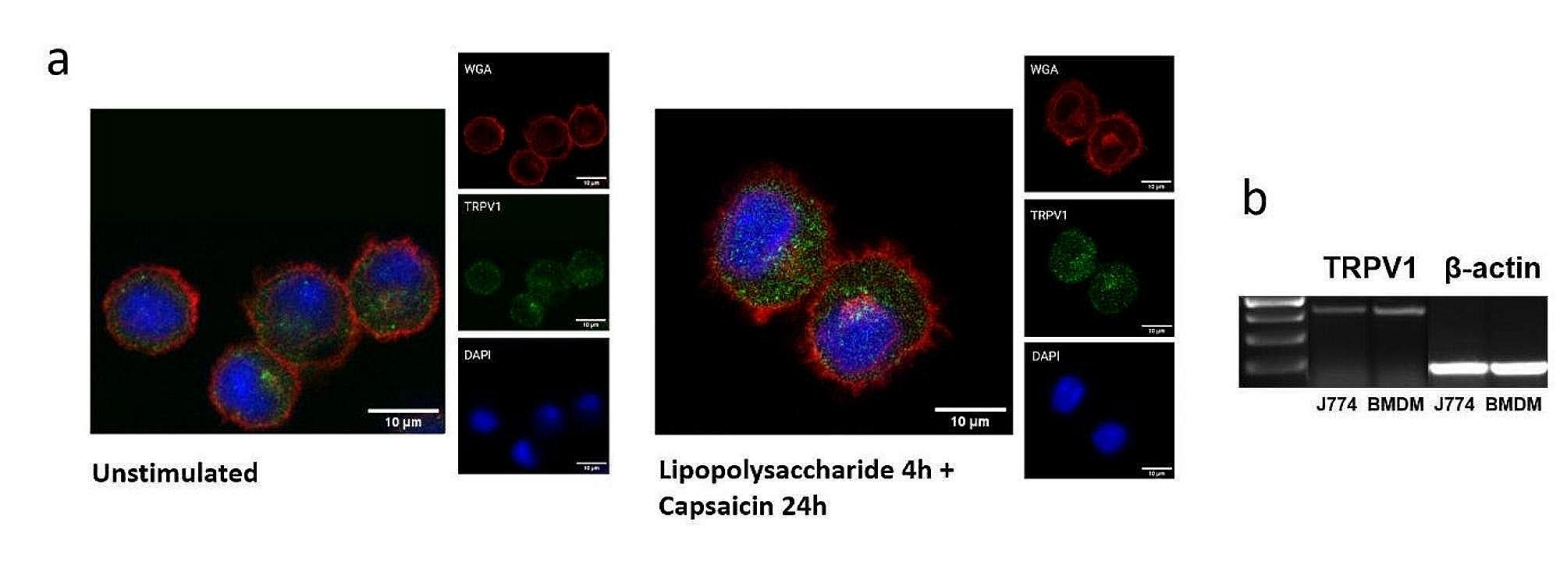
Expression of TRPV1 in murine macrophages. TRPV1 expression was documented by immunohistochemical detection in J774 cells by confocal microscopy (red – WGA 647, green – TRPV1, blue – DAPI) (**a**). Expression of mRNA level in J774 cells and bone marrow-derived macrophages (BMDM) was documented by PCR (**b**). An uncropped image of the gel is shown in Supplementary Fig. S8

To study capsaicin-induced changes in macrophage phenotype, the routinely used mouse cell line J774 and mouse BM-derived macrophages were selected [30]. The presence of TRPV1 was documented using confocal microscopy and TRPV1 mRNA expression by PCR analysis (shown in Fig. 1a, b). Full length gel. analysis of TRPV1 protein expression in the J774 cell line showed no change with the different stimulation settings (shown in Supplementary Fig. S7).

### LPS pretreatment increases capsaicin-induced calcium influx

**Fig. 2:**
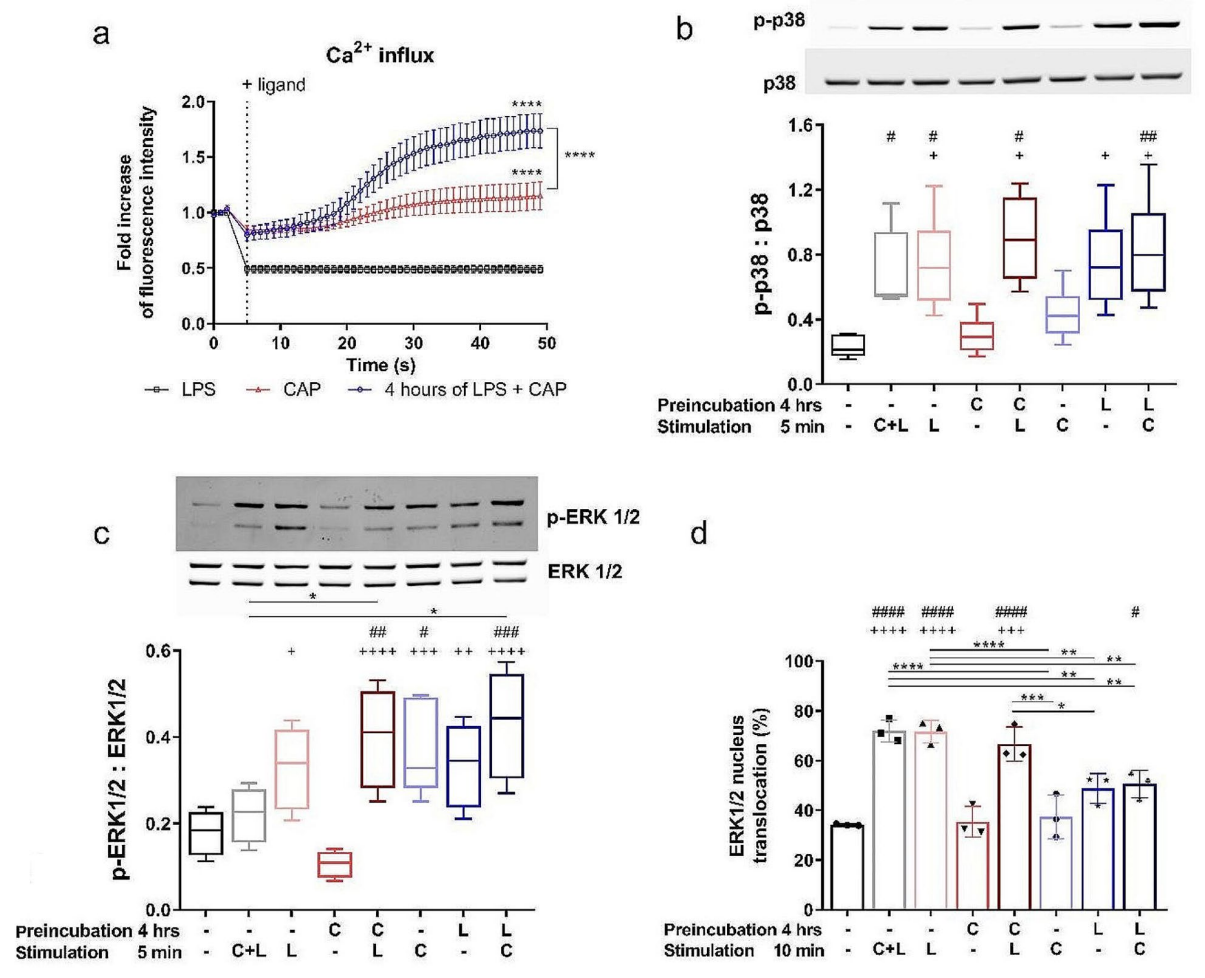
Capsaicin-induced calcium influx and modulation of LPS-induced signaling cascades. The kinetics of capsaicin-induced calcium mobilization in J774 cells were measured by the fluorescence of FLUO4-NW (**a**). Data represent mean values ± SEM. The level of statistical significance was determined using 2-way ANOVA followed by Tukey’s test for multiple comparisons (****p < 0.0001). For detection of LPS-induced signaling cascades J774 were preincubated for 4 h and stimulated with capsaicin (C) or LPS (L) for 5–10 min. The ratio between phosphorylated and unphosphorylated p-38 (**b**) and ERK 1/2 (**c**) was obtained by Western blotting after normalizing the intensities of the p-p38 and p-ERK1/2 bands to those of total p38 and ERK1/2, respectively. Uncropped images of the western blot membranes are also shown in Supplementary Fig. S9 and S10. Data are shown as boxes and whiskers. The lines within the boxes represent the median, and the whiskers represent the minimum and maximum values. *n* = 6 (**a-c**). Representative western blots are shown. The percentage of cells in which ERK1/2 translocated to the nucleus was determined by image flow cytometry. *n* = 3 (**d**). Data are shown as means ± SD. The level of statistical significance was determined using one-way ANOVA followed by Tukey’s test for multiple comparisons (^n^*p* < 0.05, ^nn^*p* < 0.01, ^nnn^*p* < 0.001, ^nnnn^*p* < 0.0001). ^#^ indicates the significance from (– –) group. ^+^ indicates significance from (C –) group. Other statistical significances between groups are indicated by *

To determine whether LPS pretreatment affects the functional potential of TRPV1 in the J774 cell line, we analyzed capsaicin-induced calcium influx in cells pretreated with and without LPS by measuring the fluorescent intensity of FLUO4-NW dye. As shown in Fig. 2a, capsaicin induced an increase in intracellular calcium levels, which was enhanced in cells pretreated with LPS for 4 h.

### Capsaicin modulates LPS-induced signaling cascades in the J774 cell line

Activation of the TRPV1 channel increases intracellular Ca^2+^ levels and activates various protein kinases, including mitogen-activated protein kinases (MAPKs), particularly ERK and p38. These MAPKs are also associated with the M2 and M1 phenotype, respectively [31]. Western blot results showed that the p38 MAPK signaling pathway was not activated in response to capsaicin, and phosphorylation of p38 MAPK was associated only with LPS stimulation (shown in Fig. 2b). On the other hand, five minutes of capsaicin stimulation significantly increased p-ERK1/2 levels compared with unstimulated J774 cells. LPS treatment activated ERK1/2 in J774 cells, but this increase was not significant compared with the control cells. An increased level of p-ERK1/2 was detected five minutes after LPS stimulation and remained elevated four hours after LPS treatment (shown in Fig. 2c). While pretreatment of J774 cells with capsaicin or LPS promoted phosphorylation of ERK1/2 induced by LPS or capsaicin, the phosphorylated form of ERK1/2 was suppressed when cells were treated with LPS and capsaicin together. Since activation of ERK1/2 signaling pathways can have a number of often contradictory effects that are cell-type and context-dependent. Several mechanisms are involved in deciding which signaling pathways are triggered, the most important of which is the cellular localization of phosphorylated ERK activity [32]. Therefore, we used Image stream flow cytometry to analyze ERK1/2 translocation into the nucleus. Interestingly, ERK1/2 nuclear translocation of each group did not correlate with ERK1/2 phosphorylation, indicating that various treatments may have different effects on the resulting macrophage phenotype (shown in Fig. 2d).

### Capsaicin modulates LPS-induced ROS and NO production by the J774 cell line

**Fig. 3:**
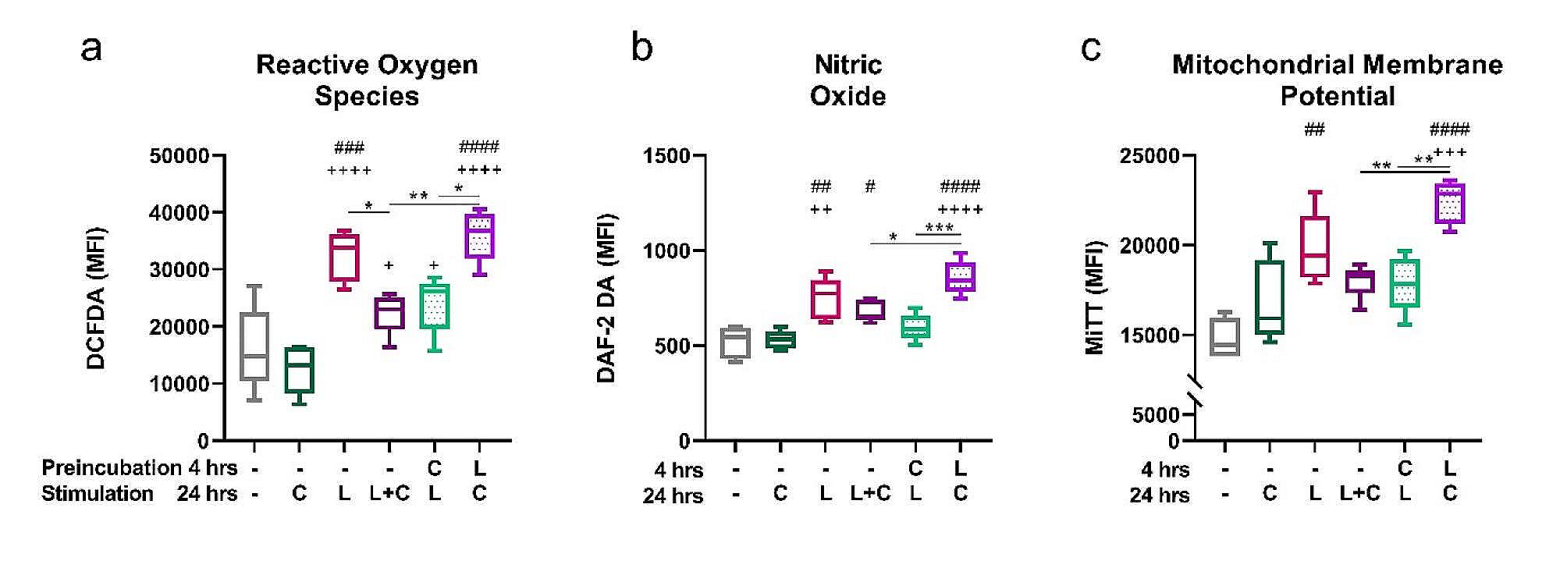
LPS-induced generation of reactive oxygen species (ROS) and nitric oxide (NO) modulated by capsaicin. J774 cells were preincubated for 4 h and cultured with capsaicin (C) or LPS (L) for 24 h. Mean fluorescence intensity (MFI) of DCFDA for the presence of ROS (**a**), DAF-2 DA for NO (**b**) and MitoTracker Red CMXRos for mitochondrial membrane potential (**c**) were determined by flow cytometry. Data are shown as boxes and whiskers. Lines inside the boxes represent the median, and whiskers represent the minimum and maximum values. *n* = 5. The level of statistical significance was determined using one-way ANOVA followed by Tukey’s test for multiple comparisons (^n^*p* < 0.05, ^nn^*p* < 0.01, ^nnn^*p* < 0.001, ^nnnn^*p* < 0.0001). # indicates significance from (– –) group. + indicates significance from (– C) group. Other statistical significances between groups are indicated by *

Macrophage activation is associated with the generation of ROS, with both M1 and M2 macrophages showing the capacity to produce ROS after activation [33]. Therefore, we determined the intracellular level of ROS and NO in the J774 cell line stimulated with capsaicin simultaneously, prior or after LPS stimulation. Although capsaicin alone did not affect ROS and NO levels, their production was increased in LPS-stimulated cells. Notably, in cells treated with LPS and capsaicin together, or pretreated with capsaicin, the mean fluorescence intensity (MFI) of DCFDA and DAF-2 DA decreased significantly, indicating lower generation of ROS and NO, while cells pretreated with LPS prior to capsaicin stimulation showed an increase in MFI (shown in Fig. 3a, b). Another important factor associated with leukocyte activation is the increase in mitochondrial membrane potential [34]. LPS-pretreated cells exhibited the highest mitochondrial potential compared to the other groups, as indicated by an increase in MFI of MitoTracker Red CMXRos (shown in Fig. 3c).

### Capsaicin modulates LPS-induced expression of MHC molecules and costimulatory receptors and the ability to stimulate T cells

**Fig. 4:**
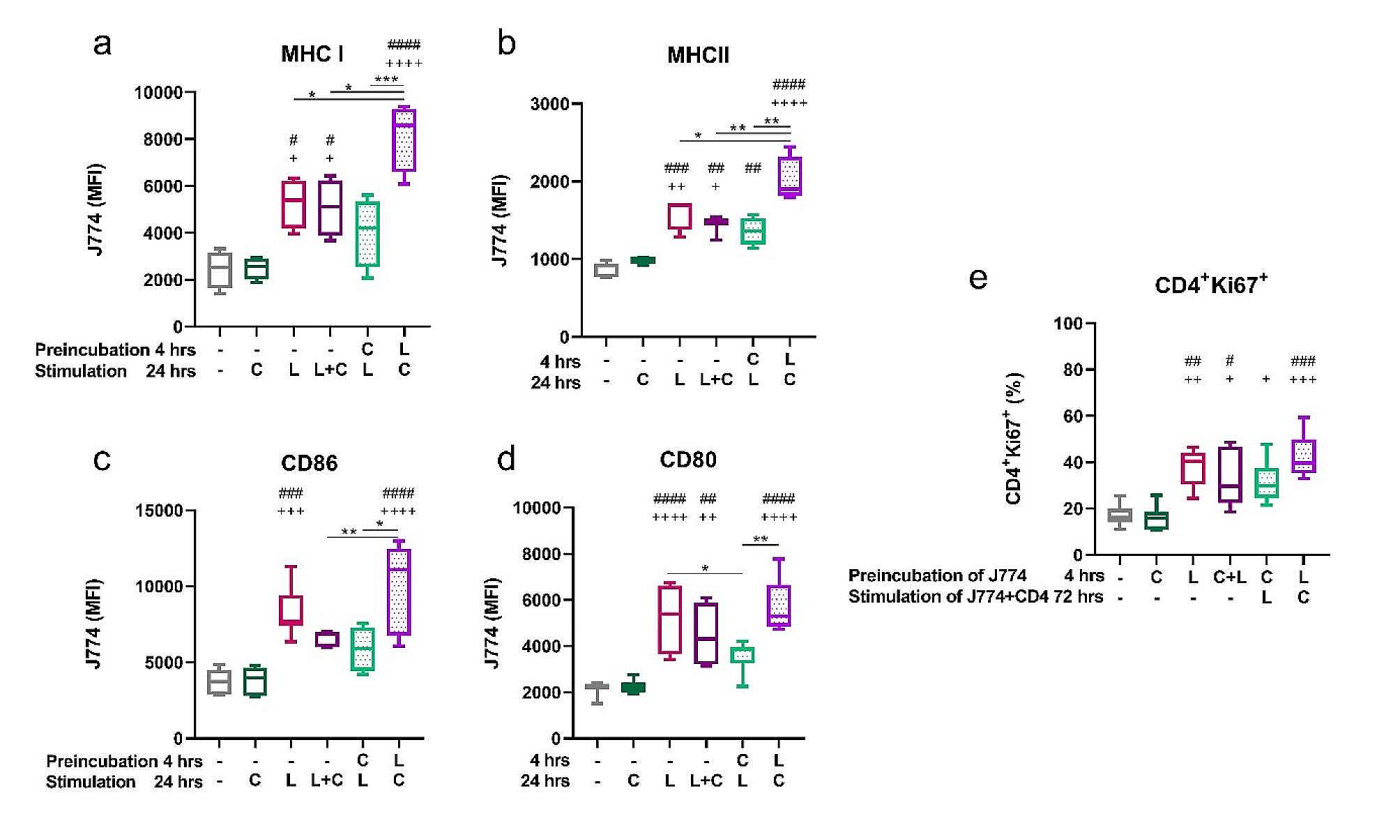
The effect of capsaicin on LPS-induced expression of costimulatory receptors and the capacity to stimulate T cells. J774 cells were preincubated for 4 h and cultured with capsaicin (C) or LPS (L) for 24 h. The MFI of MHC I (**a**) or MHC II (**b**), CD86 (c), and CD80 (**d**) of J774 was determined by flow cytometry. (**e**) The proliferation capacity of CD4^+^ T cells, was determined, according to the Ki67 expression, by flow cytometry. J774 were preincubated for 4 h with capsaicin or LPS. Afterwards, J744 and CD4^+^ (in a 1:20 ratio) were co-cultivated for 72 h in presence of capsaicin or LPS. Data are shown as boxes and whiskers. Lines inside the boxes represent the median, and whiskers represent the minimum and maximum values. *n* = 5. The level of statistical significance was determined using one-way ANOVA followed by Tukey’s test for multiple comparisons (^n^*p* < 0.05, ^nn^*p* < 0.01, ^nnn^*p* < 0.001, ^nnnn^*p* < 0.0001). ^#^ indicates significance from (– –) group. ^+^ indicates significance from (– C) group. Other statistical significances between groups are indicated by *

Subsequently, we investigated whether an increase in ROS and NO production was associated with changes in the phenotype of J774 cells. MHC molecules, and the co-stimulatory molecules CD80 and CD86 are crucially involved in macrophage activation and antigen presentation during inflammation. Indeed, the expression of MHC I and II was significantly increased in cells pretreated with LPS as compared to LPS alone, LPS and capsaicin together, and pretreatment with capsaicin (shown in Fig. 4a, b). As shown in Fig. 4c, d, pretreatment with LPS also significantly enhanced the expression of the costimulatory molecules CD86 and CD80 compared to stimulation with LPS and capsaicin together and pretreatment with capsaicin. To determine whether the increased expression of molecules associated with antigen-presenting function correlated with the capacity to stimulate T cells, CD4 positive T cells were sorted and added to the J774 cell culture in all groups studied. Indeed, the expression of Ki67, a marker of proliferating cells, was highest in T cells in coculture with LPS-pretreated J774 cells, while pretreatment with capsaicin decreased the ability of J774 macrophages to provide the co-stimulatory signal to T cells (shown in Fig. 4e).

### Capsaicin modulates LPS-induced cytokine production

**Fig. 5:**
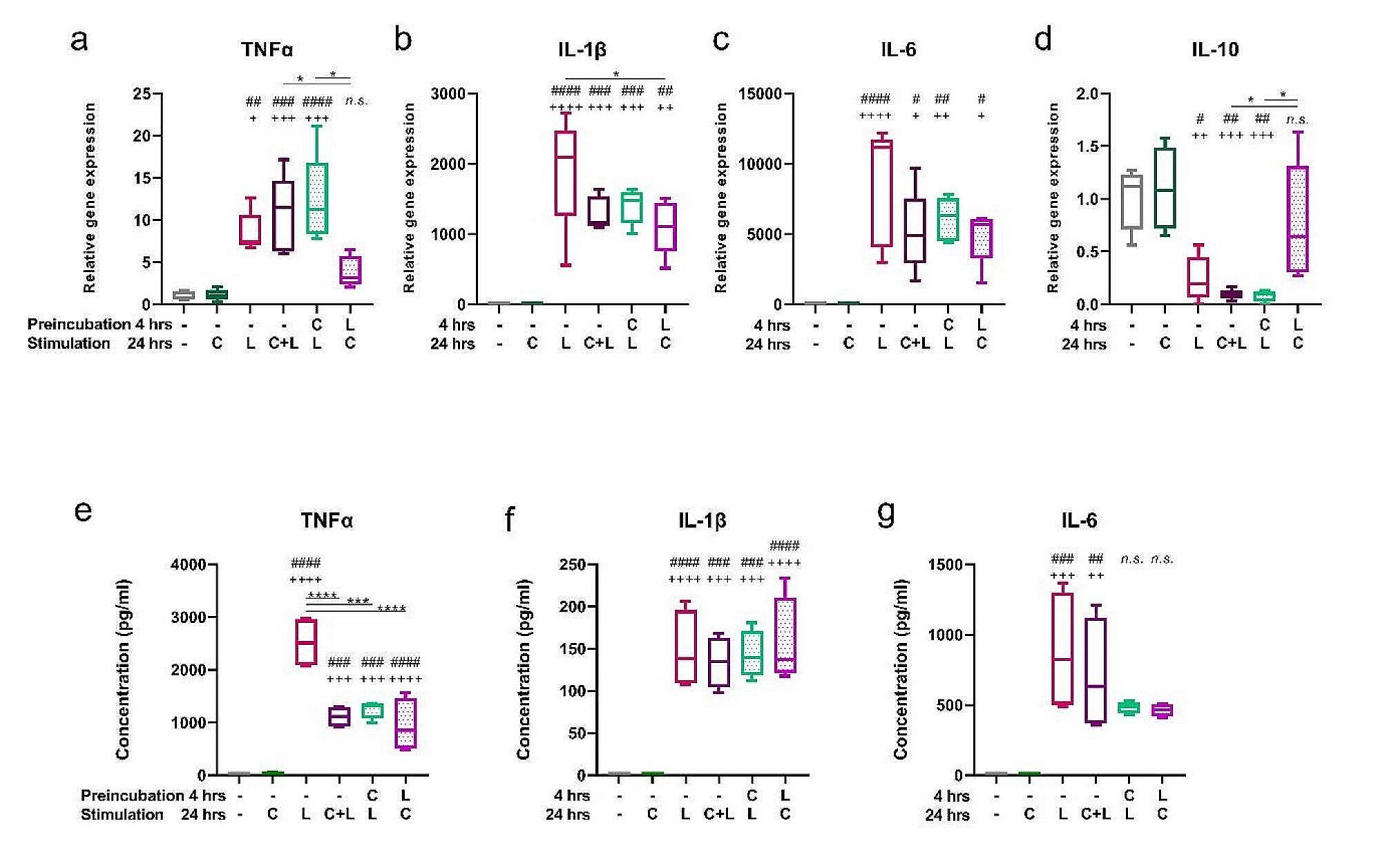
Capsaicin modulates LPS-induced cytokine production. J774 were preincubated for 4 h and cultured with capsaicin (C) or LPS (L) for 24 h. The relative gene expression of TNFα (**a**), IL-1β (**b**), IL-6 (**c**) and IL-10 (**d**) was determined by qPCR. The concentration of TNFα (**e**), IL-1β (**f**) and IL-6 (**g**) in the supernatant was determined by ELISA. Data are shown as boxes and whiskers. The lines within the boxes represent the median and whiskers represent the minimum and maximum values (**a-g**). *n* = 5 (**a-d**), *n* = 4 (**e-g**). The level of statistical significance was determined using one-way ANOVA followed by Tukey’s test for multiple comparisons (^n^*p* < 0.05, ^nn^*p* < 0.01, ^nnn^*p* < 0.001, ^nnnn^*p* < 0.0001). ^#^ indicates significance from (– –) group. ^+^ indicates significance from (– C) group. Other statistical significances between groups are indicated by *

Another important factor in determining the functional properties of macrophages is their production of cytokines. LPS stimulation of J774 cells resulted in significant expression of genes for the pro-inflammatory cytokines TNFα, IL-6 and IL-1β, which were suppressed in LPS-pretreated cells (shown in Fig. 5a, b, c). On the contrary, while the presence of LPS in the culture medium suppressed the expression of the IL-10 gene, the LPS-pretreated group showed similar expression to the control group (shown in Fig. 5d). To correlate the amount of mRNA with protein production, cytokines released into culture medium were determined by ELISA. Capsaicin treatment in all combinations reduced the concentration of TNFα in the tissue culture medium compared to the group cultured with LPS alone, but interestingly, the level of IL-1β remained similar in all groups (shown in Fig. 5e, f). IL-6 concentration was increased in LPS-stimulated cells, with both pretreatments reducing its production, although significance was not reached (shown in Fig. 5g).

### Capsaicin modulates the phenotype of bone marrow-derived macrophages

**Fig. 6:**
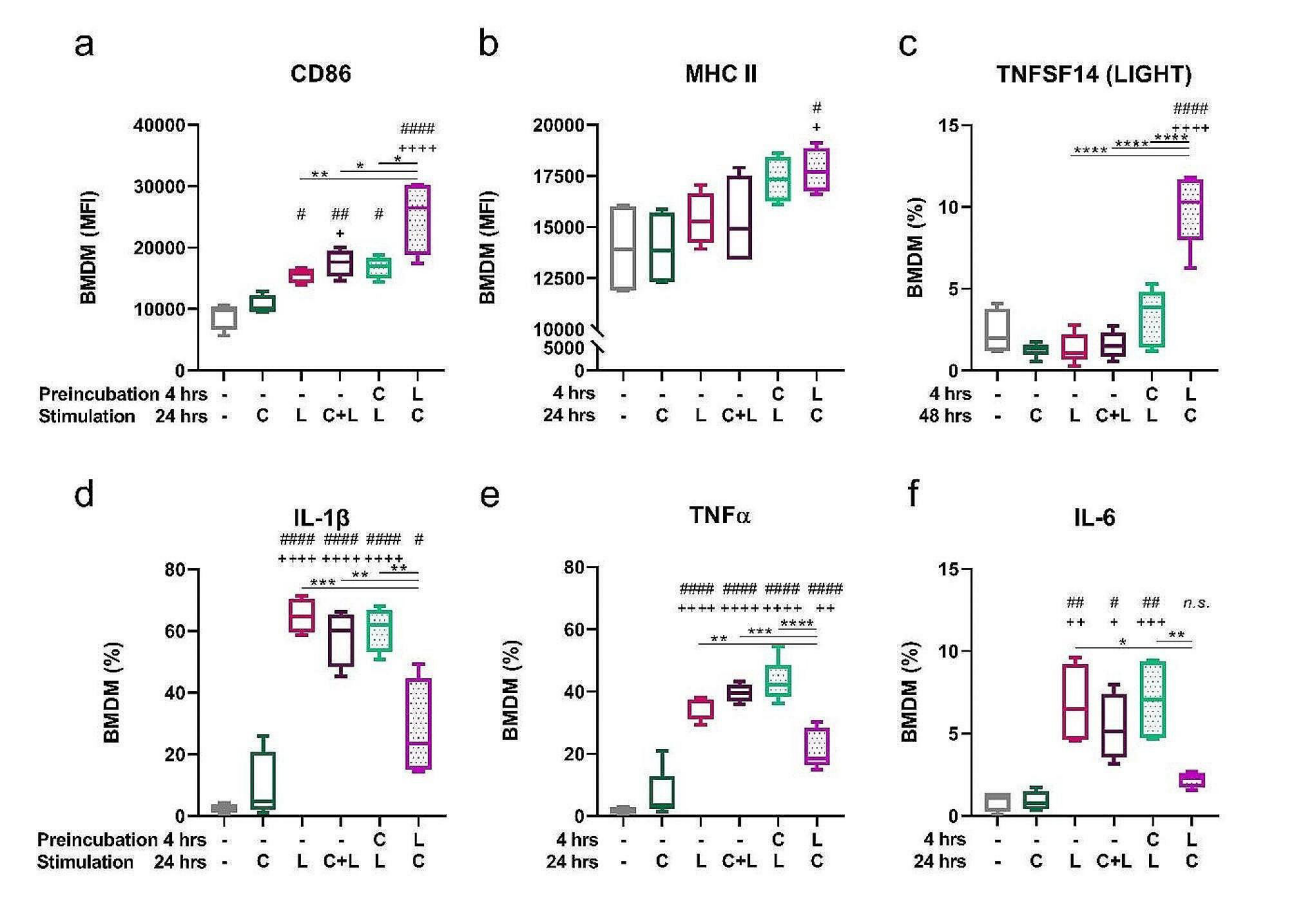
The expression of surface markers and cytokines on BM-derived macrophages. Cells were preincubated for 4 h and cultured with capsaicin (C) or LPS (L) for 24 h (48 h for TNFSF14). MFI of CD86 (**a**) or MHCII (**b**) from BM-derived macrophages and the proportion of TNFSF14^+^ (**c**), IL-1β^+^ (**d**), TNFα^+^ (**e**) and IL-6^+^ (**f**) was determined by flow cytometry. Data are shown as boxes and whiskers. Lines inside the boxes represent the median, and whiskers represent the minimum and maximum values. *n* = 5. The level of statistical significance was determined using one-way ANOVA followed by Tukey’s test for multiple comparisons (^n^*p* < 0.05, ^nn^*p* < 0.01, ^nnn^*p* < 0.001, ^nnnn^*p* < 0.0001). ^#^ indicates significance from (– –) group. ^+^ indicates significance from (– C) group. Other statistical significances between groups are indicated by *

Since the response of primary macrophage can differ from that of macrophage cell lines [30, 35], we also tested the effect of capsaicin on BM-derived macrophages. As shown in Fig. 6a, b, CD86 expression was most pronounced in the LPS-pretreated group; however, the MHC II expression was similar in all groups. M2 markers were not detectable in the J774 cell line; however, the M2b marker TNFSF14, also known as LIGHT (homologous to lymphotoxin, inducible expression, competes with herpes simplex virus [HSV] glycoprotein D for HSV entry mediator, a receptor expressed on T lymphocytes), but not the M2a and M2c markers CD206, CD163 and CD301b (shown in Supplementary Fig. S11), increased on bone marrow-derived macrophages (shown in Fig. 6c). Consistent with changes indicating the macrophage switch into an anti-inflammatory phenotype, intracellular levels of inflammatory cytokines (IL-1β, TNF-α, IL-6) decreased significantly in LPS-pretreated primary macrophages (shown in Fig. 6d, e, f).

### Capsaicin modulates the susceptibility of macrophages to *Leishmania mexicana*

**Fig. 7:**
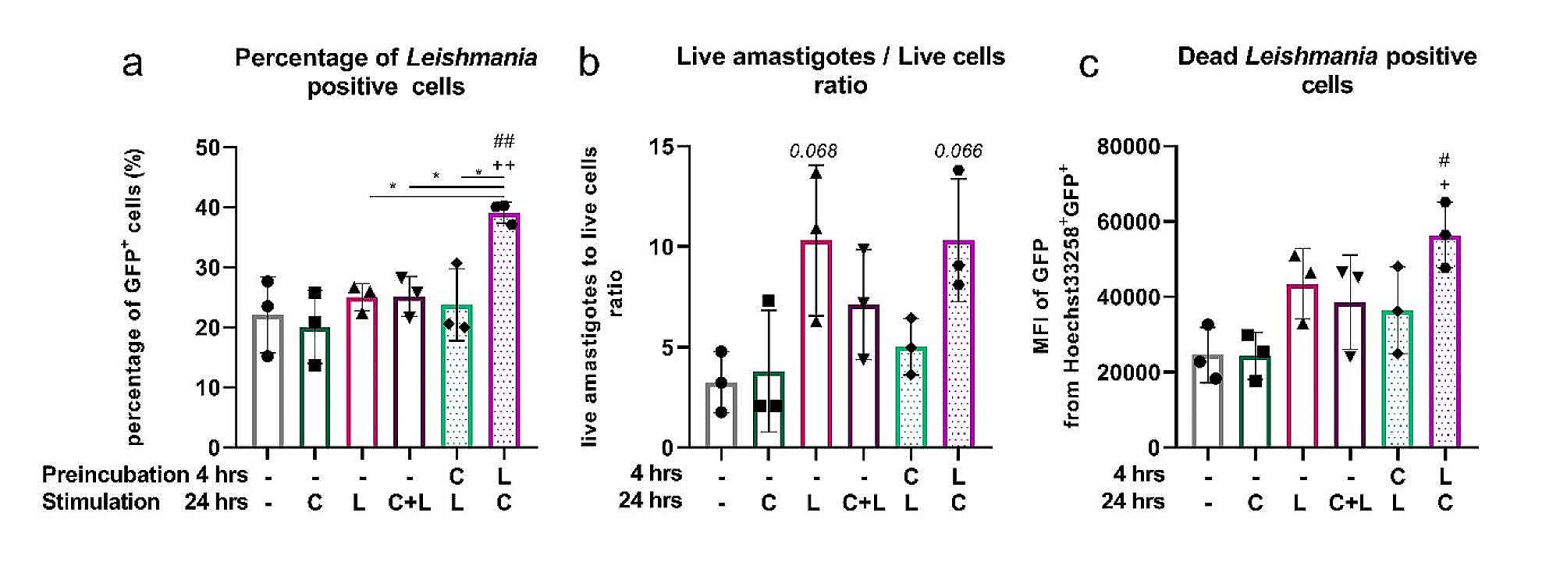
Capsaicin modulates susceptibility of J774 to *Leishmania mexicana*. J774 cells were preincubated for 4 h and cultured with capsaicin (C) or LPS (L) for 24 h. GFP-labelled *L. mexicana* promastigotes were added to cells at a ratio of 6 parasites to 1 macrophage. Percentage of *Leishmania* positive live cells was determined by flow cytometry after 72 h incubation (**a**). The ratio of live amatigotes to the number of live cells was determined by hemocytometer (**b**). MFI of GFP-labelled *L. mexicana* from dead Hoechst33258^+^ cells was determined by flow cytometry (**c**). Data are shown as means ± SD, *n* = 3 (**a-c**). The level of statistical significance was determined using one-way ANOVA followed by Tukey’s test for multiple comparisons (^n^*p* < 0.05, ^nn^*p* < 0.01). ^#^ indicates significance from (– –) group. ^+^ indicates significance from (– C) group. Other statistical significances between groups are indicated by *

Some myeloid cells serve as replication niches for the protozoan parasite *Leishmania*, and macrophages play a key role in the pathology of leishmaniasis. Individual subpopulations of macrophages contribute differently to the development of leishmaniasis, with macrophage susceptibility to *Leishmania* linked to the expression of certain M2 markers [36]. To further investigate whether capsaicin treatment affects macrophage phenotype, J774 cells were exposed to GFP-expressing *L. mexicana* [37] at a ratio of 6 parasites per 1 macrophage. Three days after infection, the percentage of *L. mexicana-*positive cells was the highest in LPS-pretreated cells (shown in Fig. 7a). In addition to different susceptibility to infection, the M2 phenotype has also been associated with the proliferation of amastigotes [36]. To address this question, the ratio of live amastigotes to the number of live cells in culture was compared between groups. The results shown in Fig. 7b indicate different proliferation of amastigotes in capsaicin- or LPS-pretreated groups, with the highest proliferation in LPS-treated cells and cells pretreated with LPS prior to capsaicin stimulation. This was associated with the highest mortality of *L. mexicana*-infected cells in this group (shown in Fig. 7c).

## Discussion

Besides its role in nociception, TRPV1 is involved in various physiological functions. Currently, the role of TRPV1 in the regulation of immune response is gaining more attention, as it has been associated with several pathological conditions [38, 39]. Rapid progress in TRPV1 channel research has provided a better understanding of its action; however, it has also produced contradictory results on the inflammatory or anti-inflammatory TRPV1 function [2, 16, 17, 22]. To explore capsaicin triggered modulation of macrophage phenotype and function by capsaicin in different inflammatory environments, we activated the J774 cell line or primary BM-derived macrophages with capsaicin simultaneously, prior to or after LPS stimulation.

Macrophages display considerable differences in their response to inflammatory stimuli depending on their origin [35], including the expression of surface molecules and cytokine production [30, 40]. Therefore, we decided to study a macrophage cell line as well as macrophages derived from the bone marrow. Indeed, some results differ in macrophages of different origins; nevertheless, combining the two types of macrophages provides a more comprehensive understanding of the role of TRPV1 in macrophage function and its inflammatory response. Importantly, both cell types express TRPV1 at mRNA and protein level which was confirmed by western blot and microscopy, using well validated antibodies [28]; therefore, providing a suitable tool for this study.

Different members of the MAPK family play distinct roles in modulating macrophage phenotype [31]. We confirmed that ERK1/2 signaling is involved in the TRPV1-mediated immune response; however, the level of phosphorylated ERK1/2 did not correlate with its translocation to the nucleus. ERK1/2 signaling has been shown to play a crucial role in many cellular processes, including proliferation, differentiation, or apoptosis, and is highly context-dependent [41]. Overall, cellular localization of activated ERK1/2 is a critical determinant of downstream signaling events; however, the effects of cellular translocation on the immune environment or cytokine production have not yet been described. Therefore, conflicting results that describe the up- or down-regulation of pro- and anti-inflammatory cytokines after ERK phosphorylation [41, 42] may be related to the differential cellular localization.

The finding that Ca^2+^ influx increased when macrophages were pretreated with LPS prior to activation with capsaicin indicated that the TRPV1 channel was sensitized by TLR4 stimulation. Sensitization of TRPV1 by LPS has been observed in sensory neurons, with the underlying mechanism involving inhibition of TLR4 activation-induced TRPV1 endocytosis [43]. Considering the expression of TLR4 on macrophages, it is reasonable to speculate that TLR4-TRPV1 interaction can occur here. The increased Ca^2+^ influx in the LPS-pretreated group was accompanied by an increase in the levels of ROS, NO and mitochondrial membrane potential, indicating that the overall macrophage activation state was increased. The increase in mitochondrial potential associated with the ROS release has been shown to play an essential role in several innate immune functions in both M1 and M2 macrophages, by regulating cell growth, differentiation, and apoptosis [44]. NO production is typically associated with M1 macrophages; nonetheless, the M2b phenotype is distinct from other M2 phenotypes due to its higher activity of iNOS and subsequent production of NO [45]. Moreover, the TRPV1 channel has been implicated in the regulation of NO release by the mechanisms mediated by the activity of NOS enzymes [46, 47]. Additionally, treatment with the NOS inhibitor L-NAME reveals the involvement of additional mechanisms. Torres-Narváez et al. (2019) proposed that capsaicin activates eNOS via Ca2^+^ influx and PI3-kinase/Akt pathways, with L-NAME selectively blocking the former [48]. Clark et al. (2007) found increased NO production in TRPV1 KO mice, suggesting that TRPV1 negatively regulates NO production upon LPS stimulation [47]. Given the known suppression of inflammatory cytokine production by NO-mediated mechanisms, NO likely plays an immunoregulatory role in the resolution of inflammation [49]. Our data suggest that the timing of TRPV1 activation in relation to LPS is critical to this phenomenon.

The definition of the different macrophage phenotypes is based on the production of specific factors, expression of cell surface markers, and biological activities. The expression of markers and the production of factors associated with M1 or M2 activation can vary depending on the stimulus and context [50]. In our study, cells stimulated with LPS prior to TRPV1 activation expressed significantly higher levels of MHC I and II as well as co-stimulatory molecules. LPS pretreated macrophages also showed the best antigen presenting function when co-cultured with naïve T cells compared with the other groups. Although M1 macrophages are generally considered to have a stronger antigen-presenting ability than M2 macrophages, the M2b subset is an exception to this rule due to the expression of co-stimulatory molecules such as CD86 and MHC class II [45, 51]. TNFSF14, which is widely accepted as an M2b marker [52], was not expressed on the J774 cell line; however, activation of TRPV1 significantly increased its expression on BM-derived macrophages pretreated with LPS. Another important feature of M2b macrophages is the reduced production of inflammatory cytokines [51]. We detected decreased expression of TNFα at the both gene and protein levels in LPS-pretreated J774 cells, whereas the expression of IL-1β remained without significant changes. It should be noted that the intracellular level of TNFα, IL-1β and IL-6 was significantly decreased in primary macrophages when they were stimulated with LPS prior to capsaicin. Significant increase in anti-inflammatory cytokines was not detected in either J774 cells or in BM-derived macrophages. According to the review by Bujak et al. (2019), decreased production of inflammatory cytokines was observed after TRPV1 activation in various in vitro models of LPS-induced inflammation [53]. However, to our knowledge, no studies have demonstrated a switch to anti-inflammatory macrophage populations producing high levels of IL-10 and low levels of inflammatory cytokines after the TRPV1 activation. Stimulation of macrophages with capsaicin prior to LPS-induced inflammation did not suppress the release of inflammatory cytokines, not only in our model but also in other studies [54]. Thus, we propose that the production of pro-inflammatory cytokines is regulated by TRPV1 activation in a manner dependent on the inflammatory milieu.

To confirm the effect of TRPV1 activation after LPS stimulation on macrophage polarization state in the functional study, we investigated the survival of GFP-labelled *L. mexicana*, an intracellular parasite that naturally infects macrophages. Polarization of macrophages has been shown to affect the course of parasitic infection [55]. In our study, LPS pretreatment of capsaicin-stimulated macrophages led to a lower level of pro-inflammatory cytokines, which can potentially create a more permissive environment for the survival and replication of *L. mexicana* within the macrophages [55]. While the sample size in our study (*n* = 3) can be considered limited, the infectious nature of the experiments presented challenges to achieving a larger sample size. Despite this limitation, our results are consistent and we believe they provide valuable insight into the survival and infection of the Leishmania parasite in macrophages. The fact that LPS treatment prior to capsaicin stimulation resulted in a higher percentage of infected macrophages, a trend toward a higher number of amastigotes per cell, and increased GFP signal intensity in dead macrophages supports our hypothesis that polarization of macrophages after TRPV1 activation is regulated by the immune microenvironment, which may also play a critical role in the outcome of parasitic infections.

## Conclusions

**Fig. 8:**
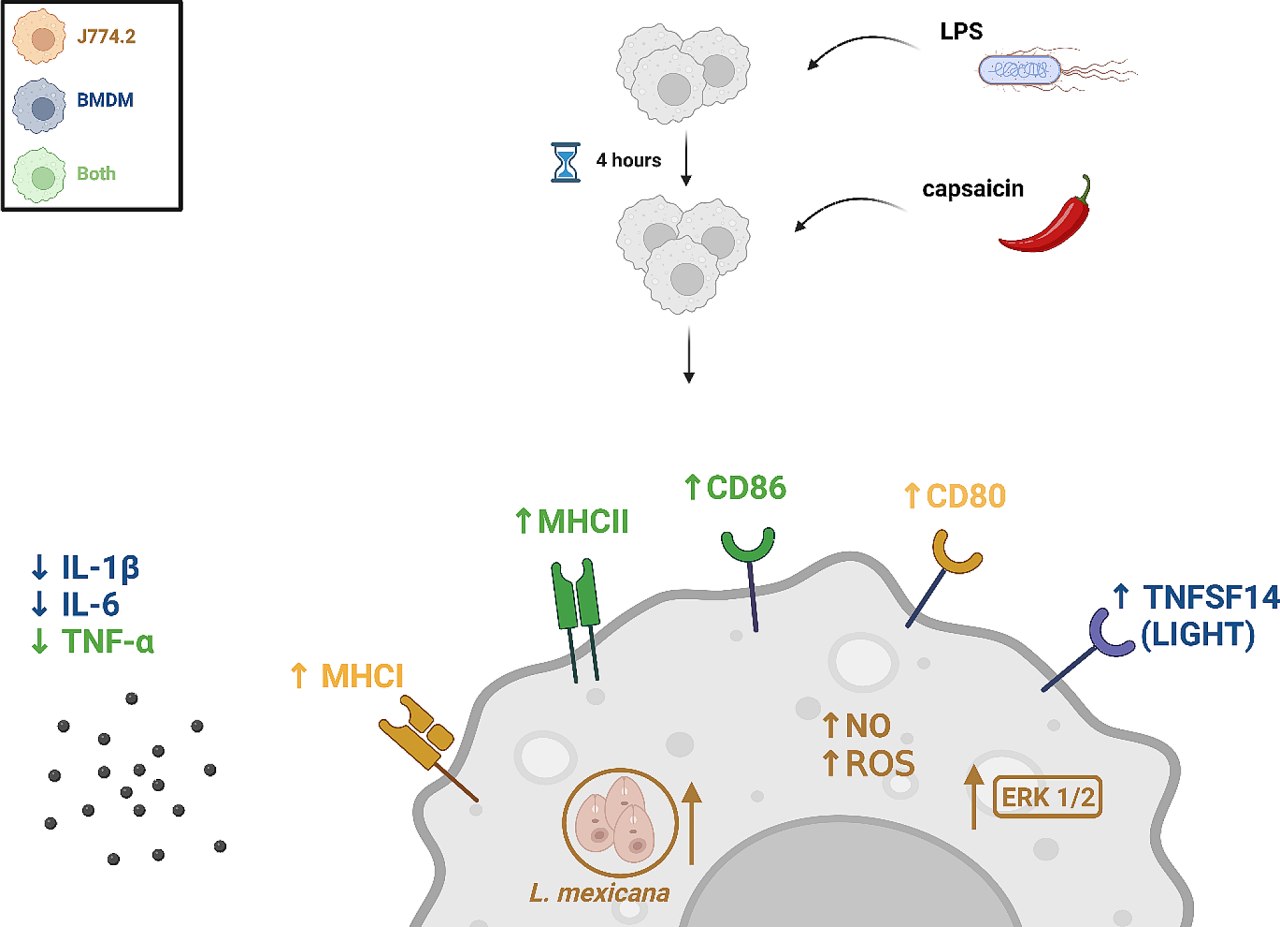
Schematic illustration of the results of this study. Macrophages pretreated with LPS and subsequently treated with capsaicin possess unique properties of M2b-like regulatory polarization state. Created with Biorender.com

In conclusion, our study shows that TRPV1 activation has specific effects on macrophages depending on the inflammatory environment, as summarized in Fig. 8. Stimulation with capsaicin of LPS-pretreated macrophages resulted in enhanced calcium influx and cell activation accompanied by a switch to the anti-inflammatory M2b-like polarization state, which was characterized by upregulated expression of MHC and co-stimulatory molecules and downregulated expression of inflammatory cytokines. ERK1/2 phosphorylation but not nuclear translocation was involved in this process. This study sheds light on the immunomodulatory mechanisms of capsaicin stimulation of macrophages in an inflammatory environment. The findings suggest that TRPV1 activation may have therapeutic potential for the treatment of inflammatory diseases by promoting an anti-inflammatory response in macrophages.

### Electronic supplementary material

Below is the link to the electronic supplementary material.

Supplementary Material 1

## Data Availability

All data generated or analysed during this study are included in this article and its supplementary material files. Further enquiries can be directed to the corresponding author.

## References

[1] Jardín I, López JJ, Diez R, Sánchez-Collado J, Cantonero C, Albarrán L (2017). TRPs Pain Sensation Front Physiol.

[2] Gouin O, L’Herondelle K, Lebonvallet N, le Gall-Ianotto C, Sakka M, Buhé V (2017). TRPV1 and TRPA1 in cutaneous neurogenic and chronic inflammation: pro-inflammatory response induced by their activation and their sensitization. Protein Cell.

[3] Lee L-Y, Gu Q (2009). Role of TRPV1 in inflammation-induced airway hypersensitivity. Curr Opin Pharmacol.

[4] Christie S, Wittert GA, Li H, Page AJ (2018). Involvement of TRPV1 channels in Energy Homeostasis. Front Endocrinol (Lausanne).

[5] Long W, Fatehi M, Soni S, Panigrahi R, Philippaert K, Yu Y (2020). Vitamin D is an endogenous partial agonist of the transient receptor potential vanilloid 1 channel. J Physiol.

[6] Yu X, Yu M, Liu Y, Yu S (2016). TRP channel functions in the gastrointestinal tract. Semin Immunopathol.

[7] Diaz-Garcia CM, Morales-Lázaro SL, Sánchez-Soto C, Velasco M, Rosenbaum T, Hiriart M (2014). Role for the TRPV1 Channel in insulin secretion from pancreatic Beta cells. J Membrane Biology 2014.

[8] Jeske NA (2015). Peripheral scaffolding and signaling pathways in Inflammatory Pain. Prog Mol Biol Transl Sci.

[9] Jara-Oseguera A, Simon SA, Rosenbaum T (2008). TRPV1: on the road to pain relief. Curr Mol Pharmacol.

[10] Andresen MC. Understanding diverse TRPV1 signaling - an update. F1000Res. 2019;8:F1000 Faculty Rev-1978. 10.12688/f1000research.20795.1 PMC6880261 31824648

[11] Luo X, Chen O, Wang Z, Bang S, Ji J, Lee SH (2021). IL-23/IL-17A/TRPV1 axis produces mechanical pain via macrophage-sensory neuron crosstalk in female mice. Neuron.

[12] Vardanyan A, Wang R, Vanderah TW, Ossipov MH, Lai J, Porreca F (2009). TRPV1 receptor in expression of Opioid-Induced Hyperalgesia. J Pain.

[13] Zhou Q, Yang L, Larson S, Basra S, Merwat S, Tan A (2016). Decreased miR-199 augments visceral pain in patients with IBS through translational upregulation of TRPV1. Gut.

[14] Billeter AT, Hellmann JL, Bhatnagar A, Polk HC (2014). Transient receptor potential ion channels: powerful regulators of cell function. Ann Surg.

[15] Caterina MJ, Julius D (2001). The Vanilloid receptor: a Molecular Gateway to the Pain Pathway. Annu Rev Neurosci.

[16] Bertin S, Aoki-Nonaka Y, de Jong PR, Nohara LL, Xu H, Stanwood SR (2014). The ion channel TRPV1 regulates the activation and proinflammatory properties of CD4 + T cells. Nat Immunol.

[17] Ninomiya Y, Tanuma SI, Tsukimoto M (2017). Differences in the effects of four TRPV1 channel antagonists on lipopolysaccharide-induced cytokine production and COX-2 expression in murine macrophages. Biochem Biophys Res Commun.

[18] Wang Y, Wang DH (2013). TRPV1 ablation aggravates inflammatory responses and organ damage during endotoxic shock. Clin Vaccine Immunol.

[19] Froghi S, Grant CR, Tandon R, Quaglia A, Davidson B, Fuller B (2021). New insights on the role of TRP Channels in Calcium Signalling and Immunomodulation: review of pathways and implications for clinical practice. Clin Rev Allergy Immunol.

[20] Amantini C, Farfariello V, Cardinali C, Morelli MB, Marinelli O, Nabissi M (2017). The TRPV1 ion channel regulates thymocyte differentiation by modulating autophagy and proteasome activity. Oncotarget.

[21] Nevius E, Srivastava PK, Basu S (2012). Oral ingestion of Capsaicin, the pungent component of Chili pepper, enhances a discreet population of macrophages and confers protection from autoimmune diabetes. Mucosal Immunol.

[22] Santoni G, Morelli MB, Amantini C, Santoni M, Nabissi M, Marinelli O (2018). Immuno-transient receptor potential Ion channels: the role in monocyte- and macrophage-mediated inflammatory responses. Front Immunol.

[23] Duo L, Wu T, Ke Z, Hu L, Wang C, Teng G (2020). Gain of function of Ion Channel TRPV1 exacerbates experimental colitis by promoting dendritic cell activation. Mol Ther Nucleic Acids.

[24] Li L, Chen C, Chiang C, Xiao T, Chen Y, Zhao Y (2021). The impact of TRPV1 on Cancer Pathogenesis and Therapy: a systematic review. Int J Biol Sci.

[25] Jiang X, Wang C, Ke Z, Duo L, Wu T, Wang W (2022). The ion channel TRPV1 gain-of-function reprograms the immune microenvironment to facilitate colorectal tumorigenesis. Cancer Lett.

[26] Liu L, Simon SA (2003). Modulation of IA currents by capsaicin in rat trigeminal ganglion neurons. J Neurophysiol.

[27] Correll CC, Phelps PT, Anthes JC, Umland S, Greenfeder S (2004). Cloning and pharmacological characterization of mouse TRPV1. Neurosci Lett.

[28] Chen J, Li L, Li Y, Liang X, Sun Q, Yu H, Zhong J, Ni Y, Chen J, Zhao Z, Gao P, Wang B, Liu D, Zhu Z, Yan Z (2015). Activation of TRPV1 channel by dietary capsaicin improves visceral fat remodeling through connexin43-mediated Ca2 + influx. Cardiovasc Diabetol.

[29] Pacakova L, Harant K, Volf P, Lestinova T (2022). Three types of Leishmania mexicana amastigotes: Proteome comparison by quantitative proteomic analysis. Front Cell Infect Microbiol.

[30] Chamberlain LM, Holt-Casper D, Gonzalez-Juarrero M, Grainger DW (2015). Extended culture of macrophages from different sources and maturation results in a common M2 phenotype. J Biomed Mater Res A.

[31] Jin Y, Liu Y, Nelin LD (2015). Extracellular signal-regulated kinase mediates expression of arginase II but not inducible nitric-oxide synthase in lipopolysaccharide-stimulated macrophages. J Biol Chem.

[32] Wainstein E, Seger R (2016). The dynamic subcellular localization of ERK: mechanisms of translocation and role in various organelles. Curr Opin Cell Biol.

[33] Lewis CV, Vinh A, Diep H, Samuel CS, Drummond GR, Kemp-Harper BK (2019). Distinct Redox Signalling following macrophage activation influences profibrotic activity. J Immunol Res.

[34] Jones AE, Divakaruni AS (2020). Macrophage activation as an archetype of mitochondrial repurposing. Mol Aspects Med.

[35] Stevens MT, Nagaria BD, Britton WJ, Saunders BM (2021). Macrophages of different tissue origin exhibit distinct inflammatory responses to mycobacterial infection. Immunol Cell Biol.

[36] Bogdan C (2020). Macrophages as host, effector and immunoregulatory cells in leishmaniasis: impact of tissue micro-environment and metabolism. Cytokine X.

[37] Podešvová L, Huang H, Yurchenko V (2017). Inducible protein stabilization system in Leishmania mexicana. Mol Biochem Parasitol.

[38] Bujak JK, Kosmala D, Szopa IM, Majchrzak K, Bednarczyk P (2019). Inflammation, Cancer and Immunity—Implication of TRPV1 Channel. Front Oncol.

[39] Xiao T, Sun M, Kang J, Zhao C (2022). Transient receptor potential Vanilloid1 (TRPV1) Channel opens Sesame of T cell responses and T cell-mediated inflammatory diseases. Front Immunol.

[40] Chamberlain LM, Godek ML, Gonzalez-Juarrero M, Grainger DW (2009). Phenotypic non-equivalence of murine (monocyte-) macrophage cells in biomaterial and inflammatory models. J Biomed Mater Res A.

[41] Shaul YD, Seger R (2007). The MEK/ERK cascade: from signaling specificity to diverse functions. Biochim Biophys Acta Mol Cell Res.

[42] Bouhamdan M, Bauerfeld C, Talreja J, Beuret L, Charron J, Samavati L (2015). MEK1 dependent and independent ERK activation regulates IL-10 and IL-12 production in bone marrow derived macrophages. Cell Signal.

[43] Min H, Cho WH, Lee H, Choi B, Kim YJ, Lee HK, et al. Association of TRPV1 and TLR4 through the TIR domain potentiates TRPV1 activity by blocking activation-induced desensitization. Mol Pain. 2018 Jan-Dec;14:1744806918812636. 10.1177/1744806918812636 PMC6856976 30355052

[44] Canton M, Sánchez-Rodríguez R, Spera I, Venegas FC, Favia M, Viola A (2021). Reactive oxygen species in macrophages: sources and targets. Front Immunol.

[45] Edwards JP, Zhang X, Frauwirth KA, Mosser DM (2006). Biochemical and functional characterization of three activated macrophage populations. J Leukoc Biol.

[46] Fernandes ES, Liang L, Smillie S-J, Kaiser F, Purcell R, Rivett DW (2012). TRPV1 deletion enhances local inflammation and accelerates the onset of systemic inflammatory response syndrome. J Immunol.

[47] Clark N, Keeble J, Fernandes ES, Starr A, Liang L, Sugden D, de Winter P, Brain SD (2007). The transient receptor potential vanilloid 1 (TRPV1) receptor protects against the onset of sepsis after endotoxin. FASEB J.

[48] Torres-Narváez JC, Pérez-Torres I, Castrejón-Téllez V, Varela-López E, Oidor-Chan VH, Guarner-Lans V, Vargas-González Á, Martínez-Memije R, Flores-Chávez P, Cervantes-Yañez EZ, Soto-Peredo CA, Pastelín-Hernández G (2019). Del Valle-Mondragón L. The role of the activation of the TRPV1 receptor and of nitric oxide in changes in endothelial and cardiac function and biomarker levels in hypertensive rats. Int J Environ Res Public Health.

[49] Kobayashi Y (2010). The regulatory role of nitric oxide in proinflammatory cytokine expression during the induction and resolution of inflammation. J Leukoc Biol.

[50] Atri C, Guerfali FZ, Laouini D (2018). Role of human macrophage polarization in inflammation during infectious diseases. Int J Mol Sci 2018.

[51] Wang Lxun, Zhang S xi, Wu Hjuan, Rong X, lu, Guo J. M2b macrophage polarization and its roles in diseases. J Leukoc Biol. 2019;106(2):345–58. 10.1002/JLB.3RU1018-378RR PMC7379745 30576000

[52] Ito I, Asai A, Suzuki S, Kobayashi M, Suzuki F (2017). M2b macrophage polarization accompanied with reduction of long noncoding RNA GAS5. Biochem Biophys Res Commun.

[53] Bujak JK, Kosmala D, Majchrzak-Kuligowska K, Bednarczyk P (2021). Functional expression of TRPV1 Ion Channel in the Canine Peripheral Blood mononuclear cells. Int J Mol Sci.

[54] Bassi MS, Gentile A, Iezzi E, Zagaglia S, Musella A, Simonelli I (2019). Transient receptor potential vanilloid 1 modulates central inflammation in multiple sclerosis. Front Neurol.

[55] Tomiotto-Pellissier F, Bortoleti BT da S, Assolini JP, Gonçalves MD, Carloto ACM, Miranda-Sapla MM, et al. Macrophage polarization in Leishmaniasis: broadening Horizons. Front Immunol. 2018;9:2529. 10.3389/fimmu.2018.02529 PMC6220043 30429856

