# Supplementary material for: Lipopolysaccharide pretreatment increases the sensitivity of the TRPV1 channel and promotes an anti-inflammatory phenotype of capsaicin-activated macrophages
Source: J Inflamm (Lond). 2024 May 24;21:17. doi: 10.1186/s12950-024-00391-0 (PMC11127439; doi:10.1186/s12950-024-00391-0)
Supplement: Supplementary file 1 — Supplementary Material 1 [file 12950_2024_391_MOESM1_ESM.docx]

**Additional file 1: Supplementary Material**

**Lipopolysaccharide pretreatment increases the sensitivity of the TRPV1 channel and promotes an anti-inflammatory phenotype of capsaicin-activated macrophages**

**Authors:**

Daniel Vašek^1+^, Natálie Fikarová^1+^, Vendula Nagy Marková^2^, Ondřej Honc^2^, Lenka Pacáková^3^, Bianka Porubská^1^, Veronika Somova^1^, Jiří Novotný^2^, Barbora Melkes^2^ and Magdaléna Krulová^1*^

**Affiliation:**

^1^ Department of Cell Biology, Faculty of Science, Charles University, Vinicna 7, Prague 2, 128 43, Czech Republic

^2^ Department of Physiology, Faculty of Science, Charles University, Vinicna 7, Prague 2, 128 43, Czech Republic

^3^ Department of Parasitology, Faculty of Science, Charles University, Vinicna 7, Prague 2, 128 43, Czech Republic

**(1 Table and 11 Figures)**

**Table S1** of used primers for RT-PCR and q-PCR.

| ***M. musculus* gene symbol** | **Official Full Name** | ***M. musculus* protein symbol** | **NCBI Gene** | **Primer sequence** | **Use** |
| --- | --- | --- | --- | --- | --- |
| ***Trpv1*** | transient receptor potential cation channel, subfamily V, member 1 | TRPV1 | 193034 | GGGTCATTTCTCCCCTACGC | RT-PCR |
|  |  |  |  | CGTAGCAACACCAGCCCAA |  |
| ***Actb*** | actin, beta | β-actin | 11461 | CATCCGTAAAGACCTCTATGCCAAC | RT-PCR |
|  |  |  |  | ATGGAGCCACCGATCCACA |  |
| ***Il1b*** | interleukin 1 beta | IL-1β | 16176 | AGTTGACGGACCCCAAAAG | q-PCR |
|  |  |  |  | AGTTGACGGACCCCAAAAG |  |
| ***Tnf*** | tumor necrosis factor | TNFα | 21926 | GCTCCAGTGAATTCGGAAAG | q-PCR |
|  |  |  |  | GATTATGGCTCAGGGTCCAA |  |
| ***Il6*** | interleukin 6 | IL-6 | 16193 | GCTACCAAACTGGATATAATCAGGA | q-PCR |
|  |  |  |  | CCAGGTAGCTATGGTACTCCAGAA |  |
| ***Il10*** | interleukin 10 | IL-10 | 16153 | AGCCGGGAAGACAATAACTG | q-PCR |
|  |  |  |  | CATTTCCGATAAGGCTTGG |  |
| ***Gapdh*** | glyceraldehyde-3-phosphate dehydrogenase | GAPDH | 16154 | CGAGGATGGGAATAACTACTG | q-PCR |
|  |  |  |  | GGATGATGA AGACAGCCTTGAAGTC |  |
| ***Actb*** | actin, beta | β-actin | 11461 | CATCCGTAAAGACCTCTATGCCAAC | q-PCR |
|  |  |  |  | ATGGAGCCACCGATCCACA |  |

**
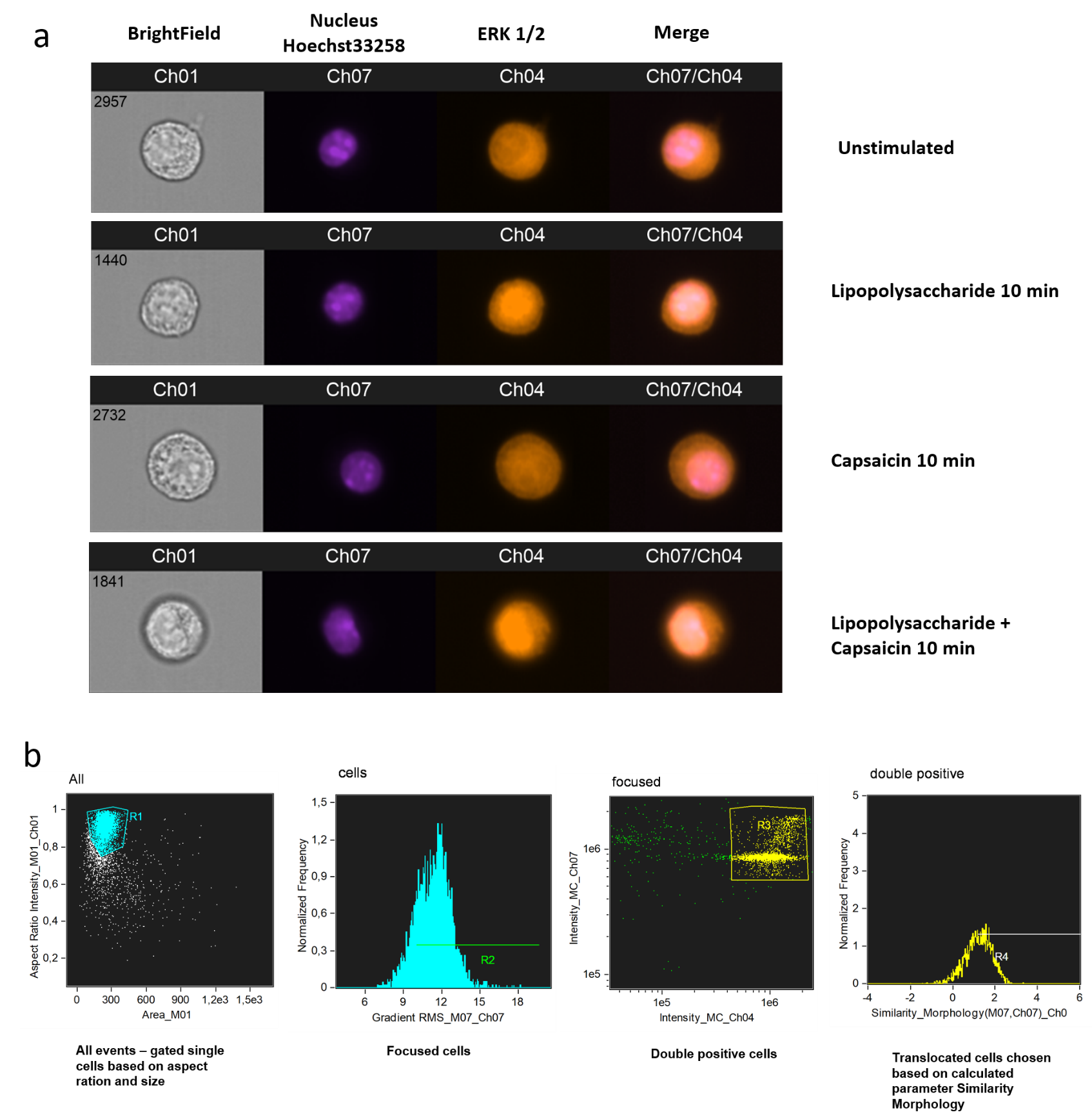
Figure S1** Gating strategy for ERK 1/2 translocation. J774 cells were preincubated for 4 hours and simulated for 10 minutes with capsaicin or/and lipopolysaccharide. Afterwards, translocation to the nuclei was determined by ImageStream flow cytometry. Example of acquired signals for individual cells (**a**). Gating strategy: after gating cells based on its size and aspect ratio (singlets determination) and focus gradient, shape of each cell and its nucleus has been determined by build-in algorithm and correlation of Hoechst 33258 signal intensity (nucleus) and AF 594 (ERK1/2) within the cell has been evaluated and Similarity Median (SM) parameter has been calculated. Region containing cells with signs of translocation has been gated on SM parameter and cells within this gate has been evaluated as „ERK translocated“ (**b**).


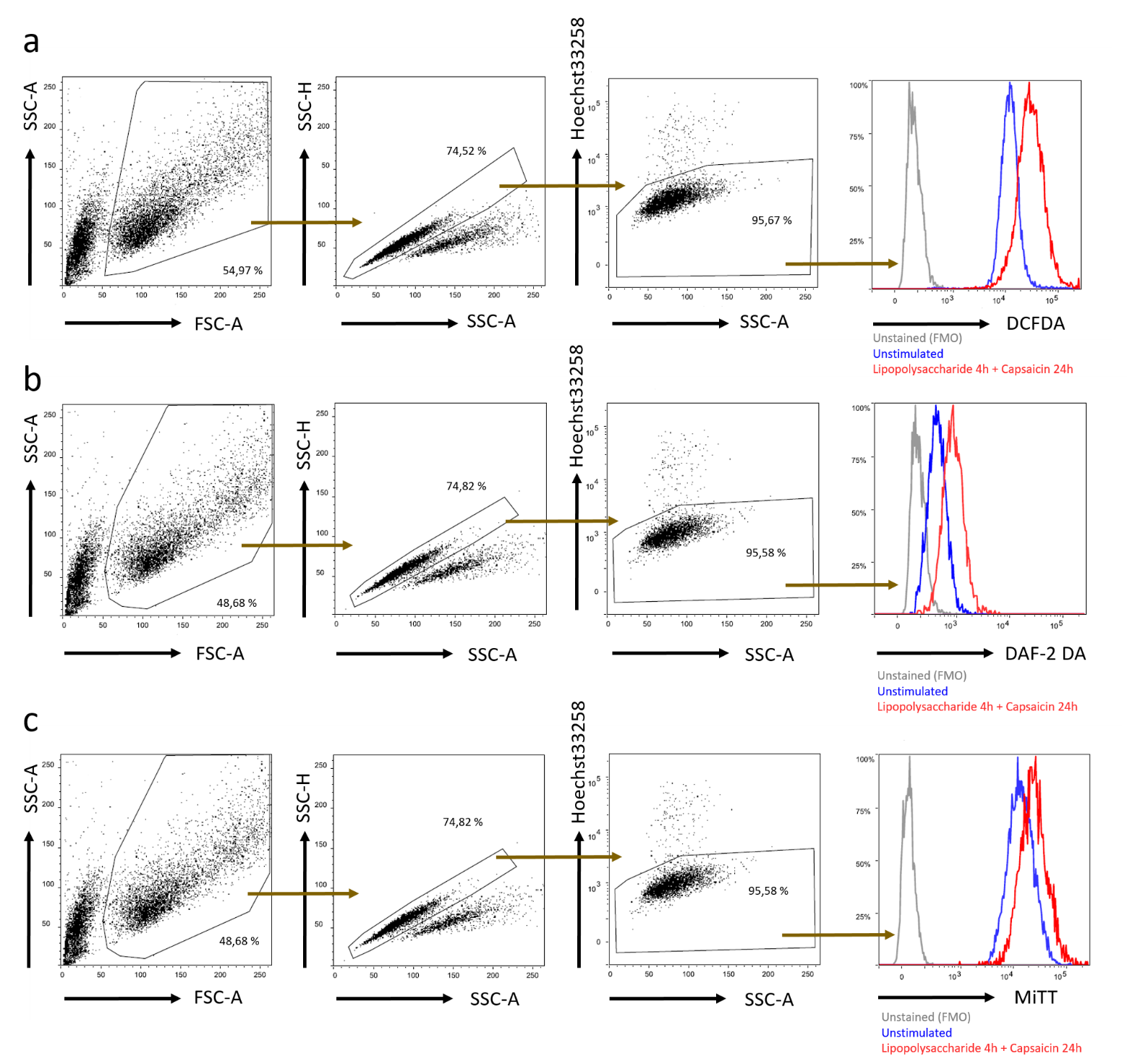


**Figure S2** Gating strategy for Reactive Oxygen Species, Intracellular Nitric Oxide and Membrane Potential of Mitochondria Staining. J774 cells were preincubated for 4 hours and simulated for 24 hours with capsaicin or/and lipopolysaccharide. Afterwards, cells were intracellularly stained for the detection of ROS production using 2′,7′-Dichlorofluorescin diacetate (DCFDA, **a**), for intracellular nitric oxide (NO) using diaminofluorescein-2 diacetate (DAF-2 DA, **b**) and for mitochondria membrane potential using MitoTracker Red CMXRos (MiTT, **c**). Gating strategy: debris was excluded based on forward and side scatter (FSC, SSC), then doublets were excluded and live were gated based on Hoechst 33258 negative population (live). From live J774 cells were determined mean fluorescent intensity for DCFDA, DAF-2 DA and MiTT. Grey – unstained FMO control, blue – unstimulated cells, red – cells preincubated with LPS 4 hrs and stimulated with capsaicin 24 hrs.


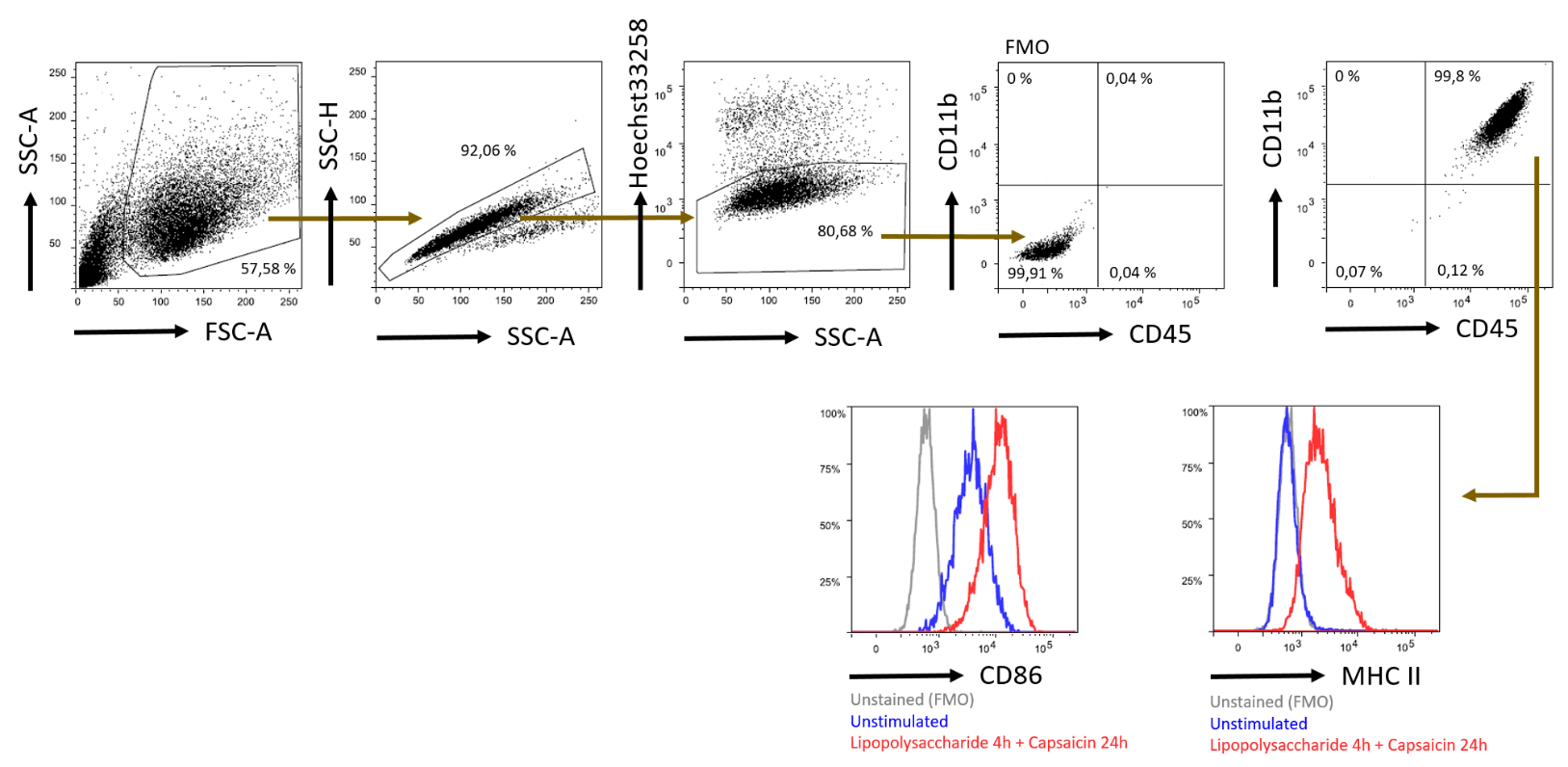


**Figure S3** Gating strategy for surface markers. J774 or BMDM were preincubated for 4 hours and simulated for 24 hours with capsaicin or/and lipopolysaccharide. Afterwards, expression of surface markers was determined by flow cytometry. Gating strategy: debris was excluded based on forward and side scatter (FSC, SSC), then doublets were excluded, and live cells were gated based on Hoechst 33258 negative population (live). Macrophages were selected based on CD45 and CD11b positivity. From CD45^+^CD11b^+^ cells were determined mean fluorescent intensity for CD80, CD86, MHC I, MHC II and percentage of TNFSF14 positive cells. Grey – unstained FMO control, blue – unstimulated cells, red – cells preincubated with LPS 4 hrs and stimulated with capsaicin 24 hrs.


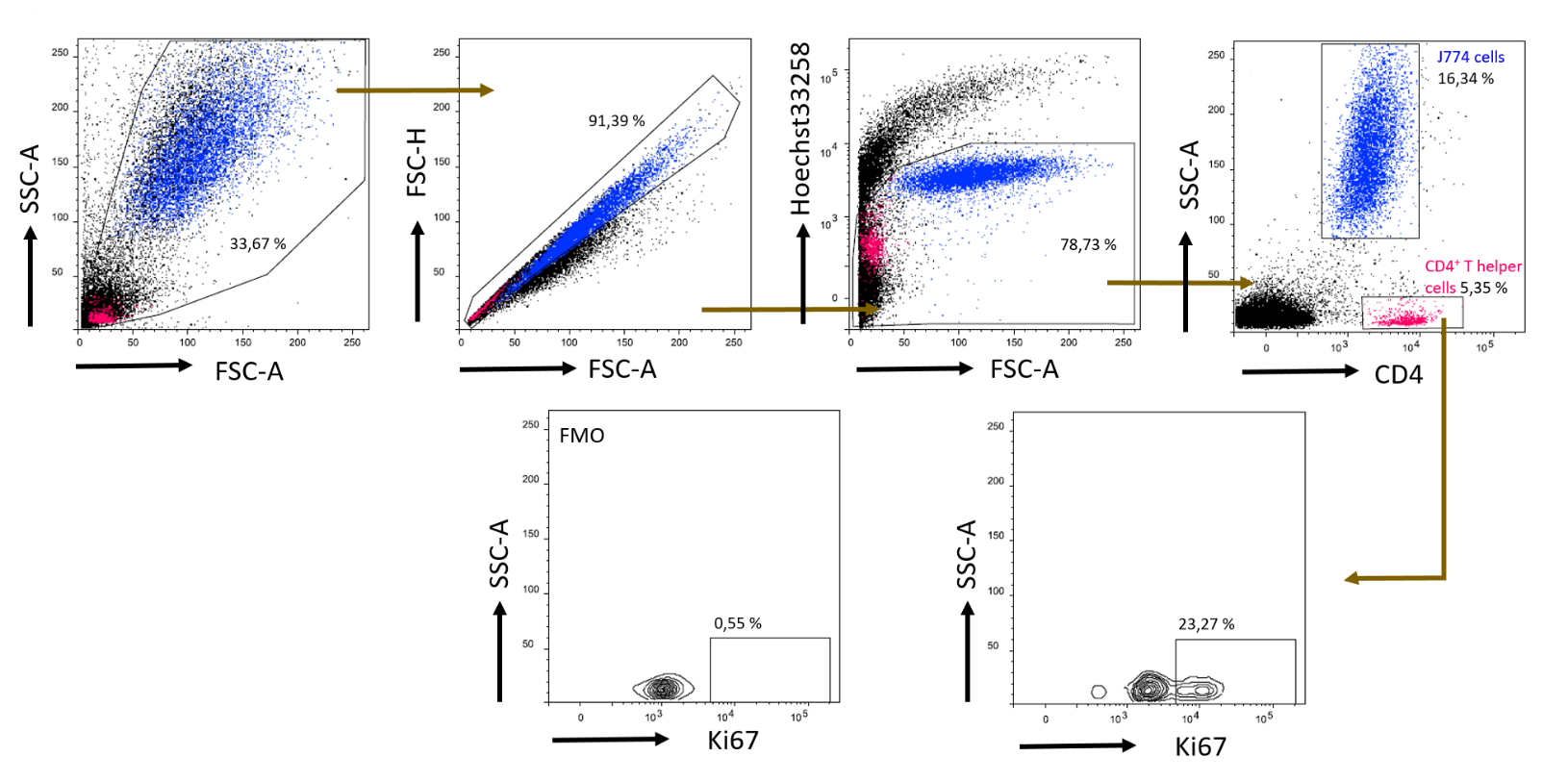


**Figure S4** Gating strategy to determine the rate of lymphocyte proliferation. Affected macrophages J774 were co-cultured for 72 hours with sorted CD4 positive cells with capsaicin or/and lipopolysaccharide. Afterwards, presence of intracellular proliferative marker Ki67 was determined by flow cytometry. Gating strategy: debris was excluded based on forward and side scatter (FSC, SSC), then doublets were excluded, and live cells were gated based on Hoechst 33258 negative population (live). T helper lymphocytes were gated based on CD4 positivity and low SSC. The back-gating was used to visualize lymphocytes and macrophages on previous dot plots (pink – CD4^+^ T helper cells, blue – J774). Proliferated T helper cells were determined by transcriptional factor Ki67. The percentage of proliferating cells (Ki67^+^) was determined from them.

**
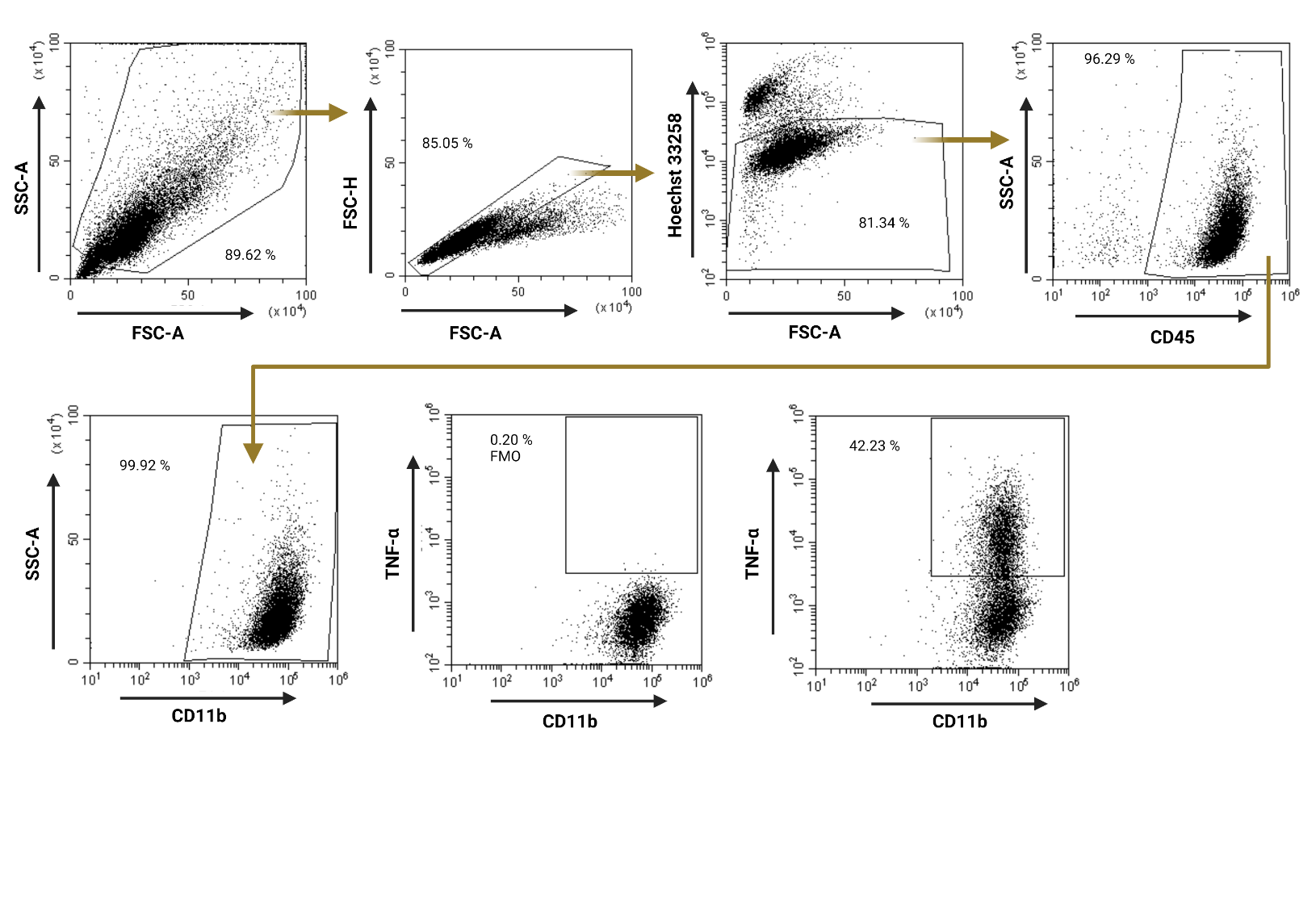
Figure S5** Gating strategy for intracellular cytokines determination. BMDM were preincubated for 4 hours and simulated for 24 hours with capsaicin or/and lipopolysaccharide. Afterwards, expression of surface markers was determined by flow cytometry. Gating strategy: debris was excluded based on forward and side scatter (FSC, SSC), then doublets were excluded, and live cells were gated based on Hoechst 33258 negative population (live). Macrophages were selected based on CD45 and CD11b positivity. From CD45^+^CD11b^+^ cells were determined percentage of TNFα, IL-1β and IL-6 cells.

**
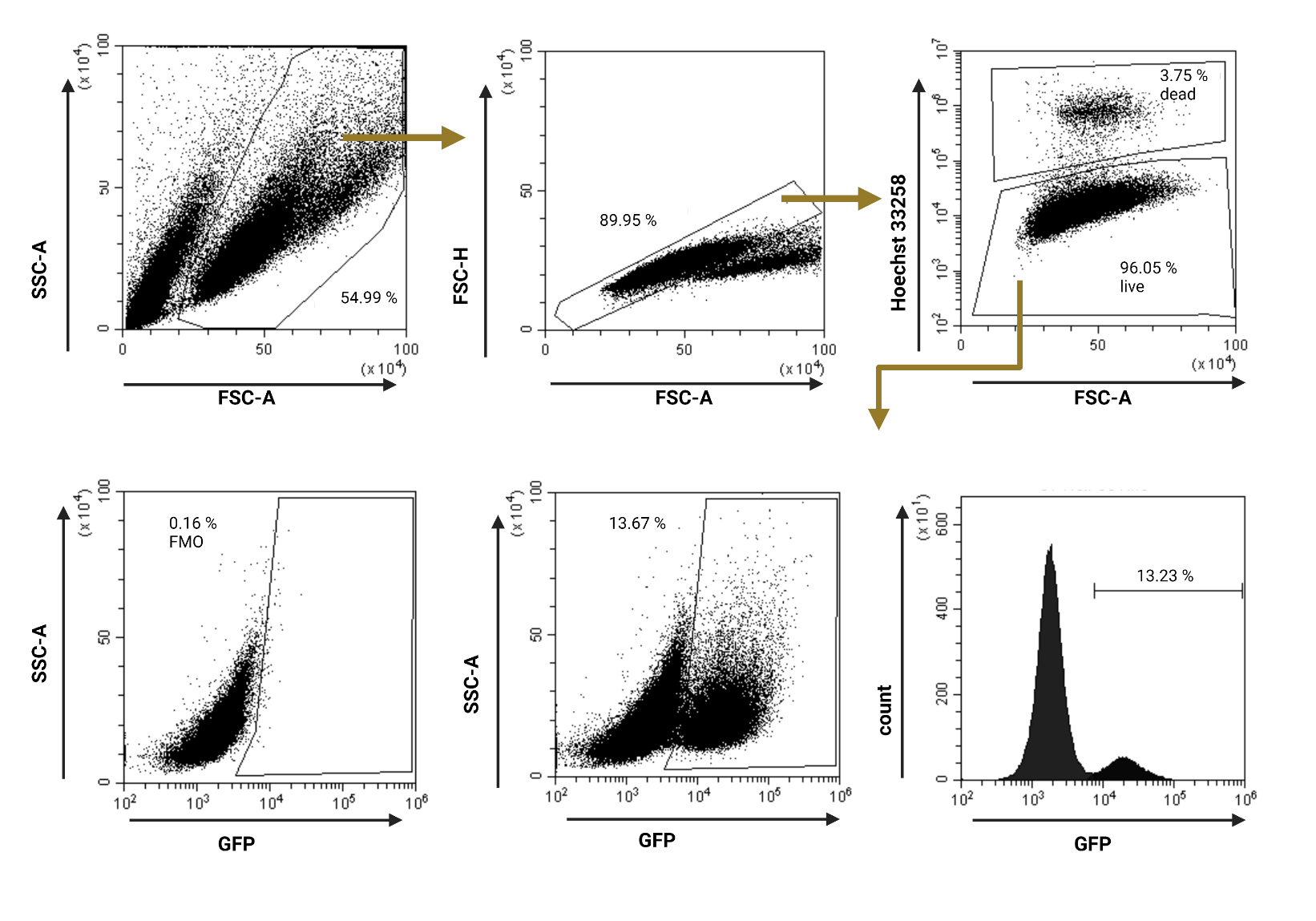
Figure S6** Gating strategy for *Leishmania mexicana* infection. GFP-labelled labelled *L. mexicana* (MNYC/BZ/62/M379) was co-cultured with affected J774 by capsaicin or/and lipopolysaccharide. Amount of phagocytosed parasites was determined by flow cytometry. Gating strategy: debris was excluded based on forward and side scatter (FSC, SSC), then doublets were excluded, and cells were divided into live and dead according to Hoechst33258 positivity. The percentage of cells positive for the GFP signal from the parasites and mean fluorescent intensity was determined.


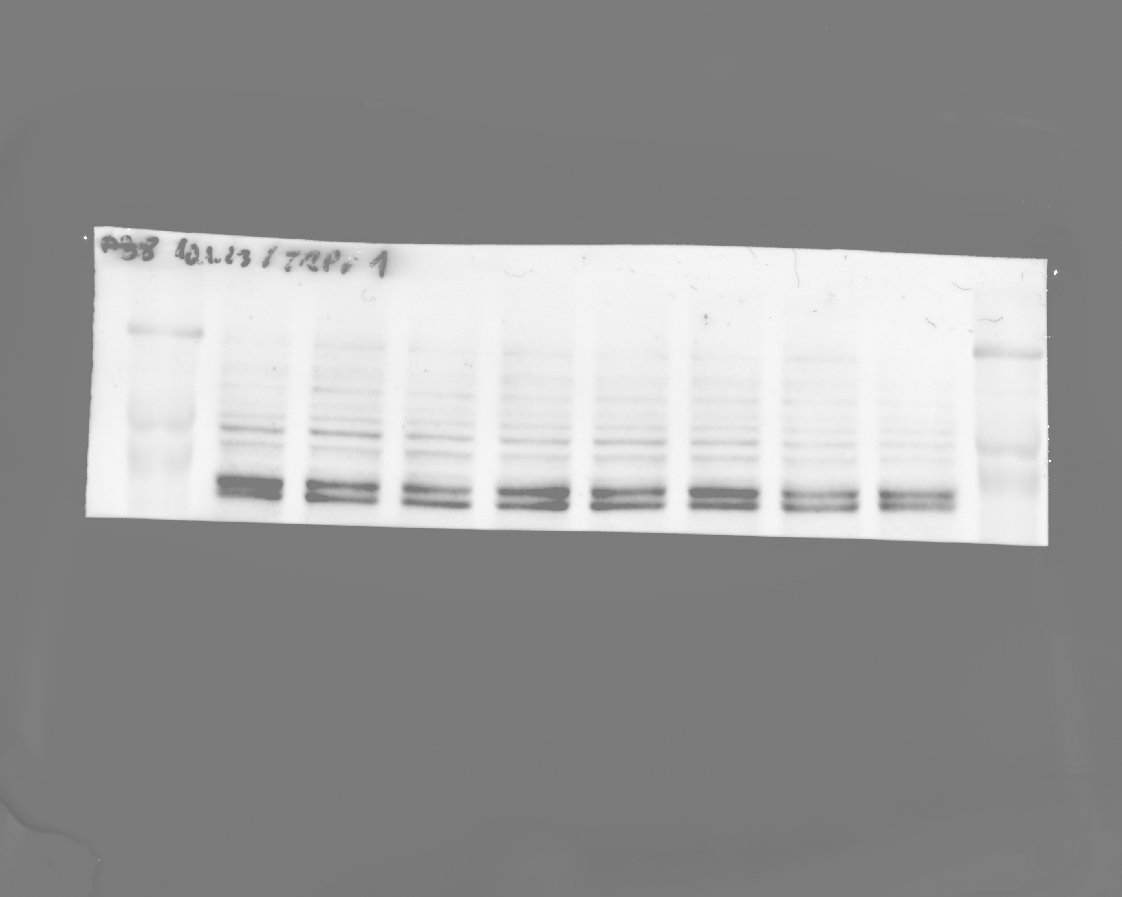

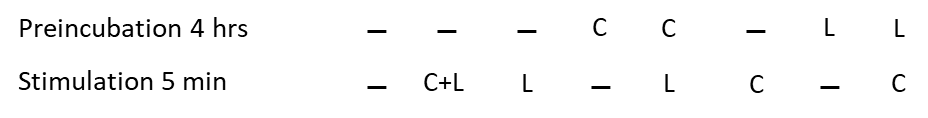


90 kDa

**Figure S7** TPRV1 protein expression. Representative Western Blot from J774 cell line preincubated for 4 hours and stimulated for 5 minutes by capsaicin (C) or lipopolysaccharide (LPS).


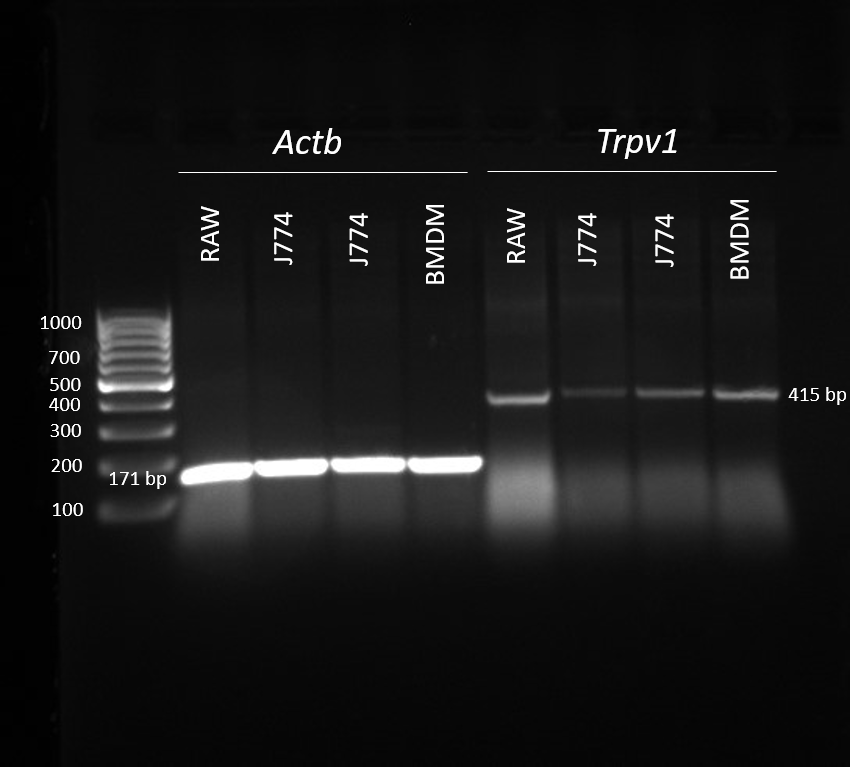


**Figure S8 *Trpv1* gene expression**. Full-length gel image of *Trpv1* and *Actb* expression. Total RNA was isolated from the J774.2 cell line (J774), bone marrow-derived macrophages (BMDM) and RAW 264.7 cell line (RAW), prepared cDNA and the presence of *Trpv1* gene expression (415 bp fragment) verified by RT-PCR. *Actb* expression (171 bp fragment) was used as a control.


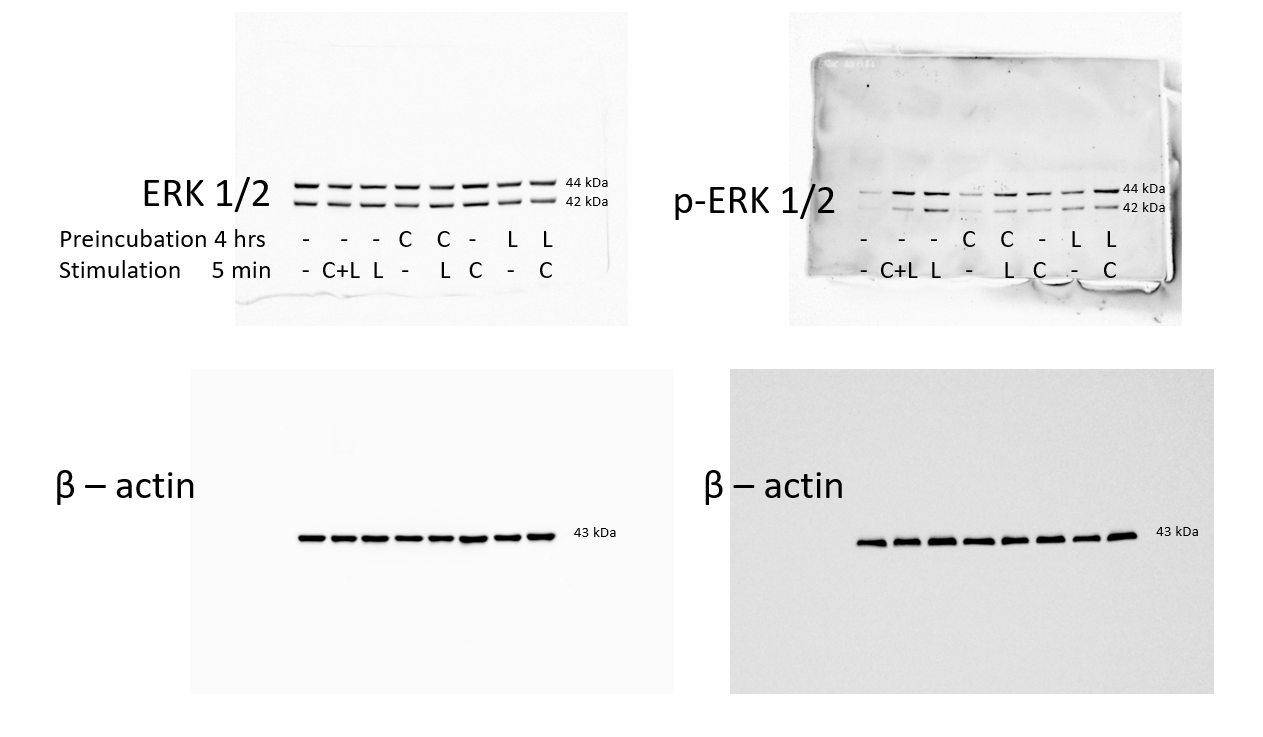


**Figure S9 ERK 1/2 protein expression.** Representative full-length Western Blot with loading control membranes from J774 cell line preincubated for 4 hours and stimulated with capsaicin (C) or LPS (L) for 5 minutes.


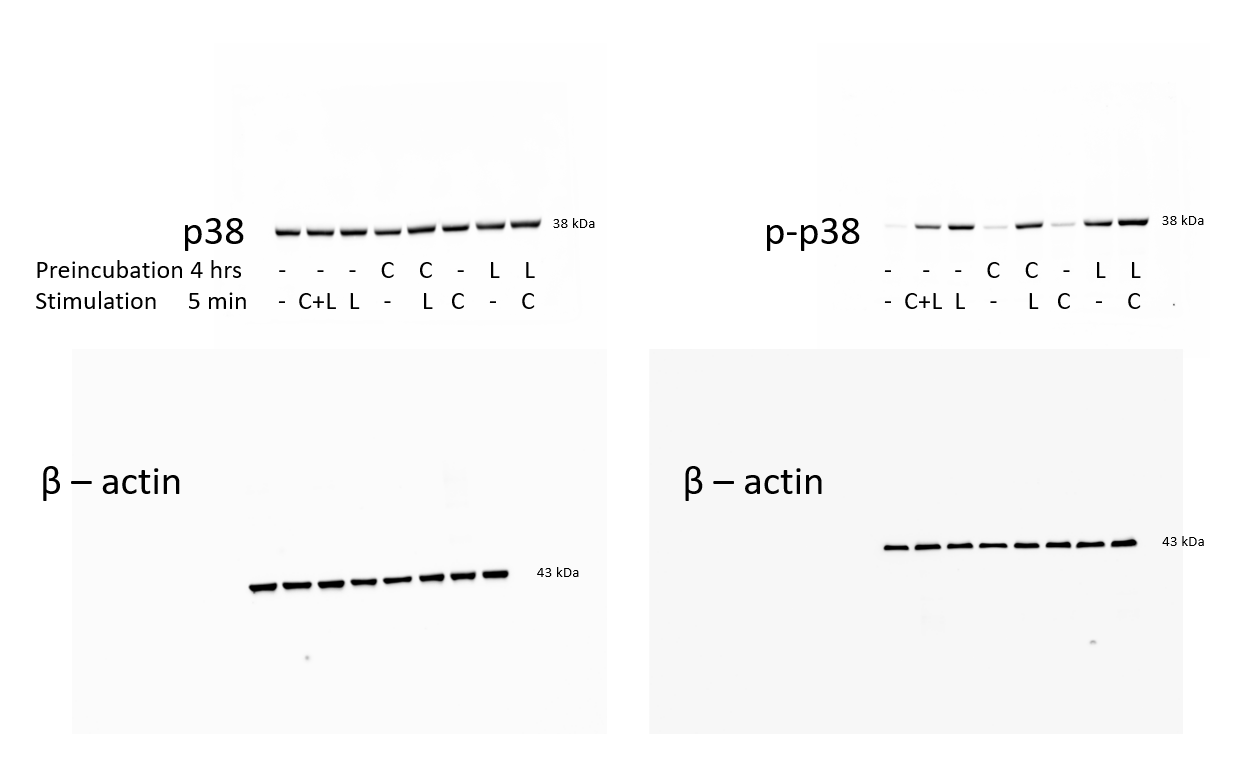

**Figure S10 p-38 protein expression.** Representative full-length Western Blot with loading control membranes from J774 cell line preincubated for 4 hours and stimulated with capsaicin (C) or LPS (L) for 5 minutes.

**Figure S11 The expression of surface markers and cytokines on BM-derived macrophages.** Cells were preincubated for 4 h and cultured with capsaicin (C) or LPS (L) for 48 h. Interleukin 4 (IL-4, 20 ng/ml) was used like positive control. Proportion of BMDM positive for CD206, CD301b (Mgl2) and CD163 was determined by flow cytometry. MFI of CD86 (**a**) or MHCII (**b**) from BM-derived macrophages and the proportion of TNFSF14^+^ (**c**), IL-1β^+^ (**d**), TNFα^+^ (**e**) and IL-6^+^ (**f**) was determined by flow cytometry. Data are shown as means ± SD from three independent experiments. The level of statistical significance was determined using one-way ANOVA followed by Tukey’s test for multiple comparisons (^n^p < 0.05, ^nn^p < 0.01, ^nnn^p < 0.001, ^nnnn^p < 0.0001). ^#^ indicates significance from (– –) group. ^+^ indicates significance from (– C) group. Other statistical significances between groups are indicated by *.
